# Momentary Depression Severity Prediction in Patients With Acute Depression Who Undergo Sleep Deprivation Therapy: Speech-Based Machine Learning Approach

**DOI:** 10.2196/64578

**Published:** 2024-12-23

**Authors:** Lisa-Marie Hartnagel, Daniel Emden, Jerome C Foo, Fabian Streit, Stephanie H Witt, Josef Frank, Matthias F Limberger, Sara E Schmitz, Maria Gilles, Marcella Rietschel, Tim Hahn, Ulrich W Ebner-Priemer, Lea Sirignano

**Affiliations:** 1 Mental mHealth Lab, Institute of Sports and Sports Science, Karlsruhe Institute of Technology, Hertzstr. 16, Building 06.31, Karlsruhe, 76187, Germany, 49 721 608 47543; 2 Medical Machine Learning Lab, Institute for Translational Psychiatry, University of Münster, Münster, Germany; 3 Department of Genetic Epidemiology in Psychiatry, Central Institute of Mental Health, Medical Faculty Mannheim / Heidelberg University, Mannheim, Germany; 4 Institute for Psychopharmacology, Central Institute of Mental Health, Medical Faculty Mannheim / Heidelberg University, Mannheim, Germany; 5 Neuroscience and Mental Health Institute, University of Alberta, Edmonton, AB, Canada; 6 Department of Psychiatry, College of Health Sciences, University of Alberta, Edmonton, AB, Canada; 7 Department of Psychiatry and Psychotherapy, Central Institute of Mental Health, Medical Faculty Mannheim / Heidelberg University, Mannheim, Germany; 8 Hector Institute for Artificial Intelligence in Psychiatry, Central Institute of Mental Health, Medical Faculty Mannheim / Heidelberg University, Mannheim, Germany

**Keywords:** ambulatory assessment, depression, speech features, openSMILE, machine learning, sleep deprivation therapy, remote monitoring, depressive disorder, mobile phone, digital health, mobile health, mHealth, mental health

## Abstract

**Background:**

Mobile devices for remote monitoring are inevitable tools to support treatment and patient care, especially in recurrent diseases such as major depressive disorder. The aim of this study was to learn if machine learning (ML) models based on longitudinal speech data are helpful in predicting momentary depression severity. Data analyses were based on a dataset including 30 inpatients during an acute depressive episode receiving sleep deprivation therapy in stationary care, an intervention inducing a rapid change in depressive symptoms in a relatively short period of time. Using an ambulatory assessment approach, we captured speech samples and assessed concomitant depression severity via self-report questionnaire over the course of 3 weeks (before, during, and after therapy). We extracted 89 speech features from the speech samples using the Extended Geneva Minimalistic Acoustic Parameter Set from the Open-Source Speech and Music Interpretation by Large-Space Extraction (audEERING) toolkit and the additional parameter speech rate.

**Objective:**

We aimed to understand if a multiparameter ML approach would significantly improve the prediction compared to previous statistical analyses, and, in addition, which mechanism for splitting training and test data was most successful, especially focusing on the idea of personalized prediction.

**Methods:**

To do so, we trained and evaluated a set of >500 ML pipelines including random forest, linear regression, support vector regression, and Extreme Gradient Boosting regression models and tested them on 5 different train-test split scenarios: a group 5-fold nested cross-validation at the subject level, a leave-one-subject-out approach, a chronological split, an odd-even split, and a random split.

**Results:**

In the 5-fold cross-validation, the leave-one-subject-out, and the chronological split approaches, none of the models were statistically different from random chance. The other two approaches produced significant results for at least one of the models tested, with similar performance. In total, the superior model was an Extreme Gradient Boosting in the odd-even split approach (*R*²=0.339, mean absolute error=0.38; both *P*<.001), indicating that 33.9% of the variance in depression severity could be predicted by the speech features.

**Conclusions:**

Overall, our analyses highlight that ML fails to predict depression scores of unseen patients, but prediction performance increased strongly compared to our previous analyses with multilevel models. We conclude that future personalized ML models might improve prediction performance even more, leading to better patient management and care.

## Introduction

Major depressive disorder (MDD) is a major global public health challenge imposing a substantial burden on individuals and society as a whole [1]. Due to the recurrent nature of MDD in many patients, relapse prevention is an important treatment goal [2]. Longitudinal symptom monitoring is crucial, especially for relapse prevention [2], as mood deterioration and prodromal symptoms can be detected in time and additional treatment can be initiated before a severe episode fully develops. However, traditional retrospective symptom questionnaires and classification interviews typically consider the last two weeks of symptoms [3], which might not be useful for the rapid detection of impending prodromal symptoms. More specifically, even an unrealistic scenario of conducting classification interviews every 2 weeks might delay the detection of a new episode by weeks [4,5]. Accordingly, approaches are needed that operate at a higher frequency, enabling us to detect prodromal symptoms, for example, on a daily basis.

Leveraging on smartphone-based data collection, promising avenues are being opened to support the traditional monitoring of MDD symptoms [6,7]. Offering continuous, unobtrusive, near–real-time, active and passive everyday life data collection, the use of ambulatory assessment (AA) increases ecologically valid insights into the lives of people living with mental disorders [8,9]. Widespread personal digital devices such as smartphones are used to capture momentary self-reported symptoms and behaviors as patients go about their normal daily activities in their natural environment [10]. As clear biomarkers for MDD are lacking [11], the identification of behavioral markers that can be objectively derived from digitally captured everyday life behavior has great potential to increase automated detection of new episodes, ultimately improving depression care [6,12,13].

Speech has been discussed as one such potential behavioral marker [14]. As early as 1921, Kraepelin [15] observed that patients with MDD tended to speak with a lower speech rate, more monotonously, and at a lower pitch compared to healthy individuals. Since then, many studies have described further depression-related altered speech characteristics [14,16]. However, the research field faces several challenges such as the sheer limitless volume of potential speech features. Inference statistics require a theory-driven selection of parameters, as combining thousands of them increases the α error [17]. Machine learning (ML) techniques offer a data-driven alternative, allowing a variety of parameters to be explored without the need for a priori parameter restriction.

Most studies investigating speech in MDD (independent of using ML or classical inferential statistics) use case-control designs, comparing speech samples (or often a single sample) of patients with MDD to healthy controls [14]. While this approach is initially useful, it does not address the prediction of upcoming episodes. To predict new emerging episodes or prodromal symptoms, we need patient data before an episode and during an emerging episode with prodromal symptoms; even better is to collect data during and after an episode. Such data would allow us to train models to discriminate between healthy, prodromal, and disordered states on a within-person level or to relate speech features to dimensional symptomatology. This would approximate the ultimate goal in clinical practice, namely to decide within a given patient that yesterday’s speech features were normal, but today’s speech features predict an emerging episode. Unfortunately, longitudinal studies of patients with MDD including regular speech samples, regular psychopathological ratings as ground truth and sufficient variance in this ground truth, that is, changes in healthy and disordered states, are rare [14,16].

To address this gap, we used a longitudinal dataset in which repeated assessments of depressive momentary states and speech features derived from selfie videos were collected concomitantly by patients with an acute depressive episode [18]. While Wadle and colleagues [18] used classical statistics (multilevel models) and focused on 3 specific, theory-driven speech features (speech rate, speech pauses, and pitch variability), which did indeed show associations with depression severity, we wanted to improve on several levels. Given the large number of speech features available, the aim of this study was to extend our previous findings by examining a comprehensive set of 89 speech features and by using more complex modeling approaches in terms of ML. We aim to contribute to this field, as we only identified 3 ML studies using longitudinally assessed data in a clinical (as opposed to subclinical) population with multiple data points per patient to predict depression severity based on speech features.

In one of the studies, speech samples and concomitant mood self-ratings were collected from 30 patients with MDD via AA over the course of 2 weeks [19]. ML analyses revealed a correlation of ρ=.61 between the actual and predicted mood scores, and an improvement in prediction when using personalized (ρ=.79) instead of nonpersonalized models.

The most promising dataset at present is from the consortium of the Remote Assessment of Disease and Relapse—Central Nervous System (RADAR—CNS) project, with 2 relevant publications [20,21]. In the study by Cummins et al [21], speech data were collected in the form of a scripted task and a free-response task from 461 patients with MDD every 2 weeks for 18 months. A set of 28 speech features was analyzed using linear mixed models. Associations were found between elevated depression symptoms and speech rate, articulation rate, and speech intensity. However, the authors mention in their limitations that the results are based on the cohort level, which limits insights into intraindividual depression-related speech changes, which they plan to investigate in the future. The other publication from the RADAR—CNS project focused on the benefits of model personalization [20]. Data from the scripted (n=271) and free response (n=258) task from a subset of patients were used to explore personalized and generalized ML models. Three speech parameter sets were extracted from a total of 8004 speech samples, with personalization proving beneficial for their binary depression classification (high or low depression severity). Specifically, running a support vector regression (SVR) classifier based on the extended version of the Geneva Minimalistic Acoustic Parameter Set (eGeMAPS, audEERING) from the free-response task for this binary decision resulted in better performance for the personalized compared to the generalized models.

Building on previous work by the authors [18], we aim to contribute to closing this gap and to the understanding of speech-based longitudinal monitoring of MDD. Specifically, we were interested in whether a multiparameter ML approach would significantly improve prediction compared to our previous study, which focused on the three most prominent speech features. In addition, we explored which mechanism for splitting training and test data was most successful, with a particular focus on the idea of personalized prediction. To do so, we analyzed a dataset of patients (n=30) diagnosed with MDD during sleep deprivation therapy, a fast-acting treatment that results in a significant improvement of depressive states in most of the patients within 36 hours [22]. The given treatment ensures short-term effects, which is advantageous compared to other studies such as the RADAR—CNS project where patients are observed for over 2 years to reveal illness episodes [23]. In Wadle et al [18], patients reported momentary depressive states and recorded concomitant selfie videos talking about their current feelings 2‐3 times per day for up to 3 weeks. Speech features were extracted from the speech samples using the software openSMILE [Open-Source Speech and Music Interpretation by Large-Space Extraction]) [24]. To assess the potential clinical utility of automated symptom monitoring using speech features, we trained and evaluated a comprehensive set of >500 ML pipelines (by optimizing hyperparameters of random chance and dummy regressors for baseline comparisons, random forest, linear regression, SVR, and XGBoost [Extreme Gradient Boosting] regression models) to predict individual symptom severity. We used five different approaches to evaluate whether these ML models generalize across patients or whether personalized splits are superior: (1) group 5-fold cross-validation at the subject level; (2) a leave-one-subject-out (LOSO) approach; and (3) a train-test-split with 2-fold cross-validation using different splitting techniques: (3a) chronological split with the first half as training and the second half as test set; (3b) odd-even split, with chronologically sorted data put into train and test set by turns; and (3c) a random split, which was repeated 10 times.

## Methods

### Sample

We analyzed a dataset that was collected as part of the Sleep Deprivation and Gene Expression II pilot study (DRKS00022025). The initial sample consisted of 30 inpatients from the Central Institute of Mental Health in Mannheim, Germany, who experienced an acute depressive episode as defined in the *ICD-10* (*International Statistical Classification of Diseases, Tenth Revision*) on admission to the hospital. The final sample to be analyzed consisted of 22 (n=12, 55% male) patients aged between 18 and 63 (mean 33.5, SD 12.4, median 29, IQR 23.25-42.75) years, as the dataset of 8 patients had to be excluded completely. Specifically, 4 patients did not record any videos, 1 patient did not say anything during the recordings (23 videos), the data of 2 patients lacked sound due to technical problems (30 videos), and 1 patient was excluded because they recorded only 2 videos. The final sample corresponds to 18 patients with moderate depression and 4 patients with severe depression at study inclusion as assessed by clinical expert interviews using the Montgomery-Åsberg Depression Rating Scale [25]. The mean score was 28 for patients with moderate depression and 39 points for patients with severe depression. Exclusion criteria were comorbid substance use disorders and personality disorders.

### Study Procedure

**Figure 1.**
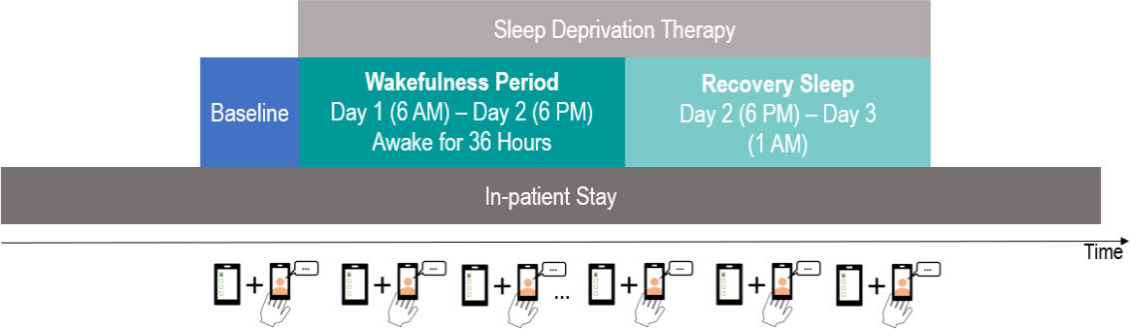
Study design.

Data were collected by patients on a study smartphone using the movisensXS software (movisens GmbH). The patients underwent sleep deprivation therapy as part of their depression treatment. In other words, patients had to stay awake for approximately 36 hours. Treatment effect and relapse can be measured in a matter of 4 days [22], resulting in substantial within-person variance for many patients in the dataset. After at least 1 day of baseline assessment, sleep deprivation therapy was conducted on what we define as day 1 (Figure 1). Specifically, patients stayed awake from 6 AM on day 1 to 6 PM on day 2. Recovery sleep was allowed from 6 PM on day 2 until 1 AM on day 3. Data were collected before, during, and after sleep deprivation therapy for up to 26 days. During the first week of this study, smartphones sent prompts three times per day (morning, afternoon, and evening); in addition, self-initiated assessments were possible to report specific events or to catch up on missed assessments. To reduce patient burden, the sampling scheme was changed to two prompts per day (morning and evening). At each prompt, patients were asked to complete items about their current affective state and to record a selfie video reporting how they currently felt. Patients returned the smartphone at the end of this study.

### AA: e-Diary Ratings and Selfie Videos

The dataset contains three sets of momentary affect ratings in the form of e-diary ratings at each prompt. The full assessment tools are described in Wadle et al [18]. As the analysis in this work is limited to the target variable of momentary depression, we focus here on its detailed description. Depression severity was assessed using the short version of the Allgemeine Depressionsskala (ADS-K) [26]. We adapted the ADS-K to fit the characteristics of momentary assessment with 14 items on depressive mood (excluding the sleep item) rated on a scale from 0=*rarely* to 3=*mostly* (Multimedia Appendix 1). We recoded the reversed items so that higher scores indicated higher intensity of depressive symptoms, thereafter, we calculated mean values. In addition to the e-diary ratings just described, patients were asked to record selfie videos with the following instructions: “Please keep the camera stable during the recording and record your whole face. Please describe in 10‐20 seconds how you currently feel.”

### Ethical Considerations

The Ethics Committee II of the Medical Faculty Mannheim, University of Heidelberg, Germany, approved this study (2013-563N-MA). Patients were informed about the aims and study procedures. All patients gave informed consent and could withdraw from this study at any time.

### Data Preprocessing

#### Overview

Initially, the dataset contained 899 recorded selfie videos. As mentioned above, we excluded all videos of 4 patients (55 videos) and removed 2 additional videos with technical damage. We extracted audio tracks from the 842 remaining videos using the *ffmpeg* package in Python (Python Software Foundation) and archived them as .wav files (sampling rate=48 kHz, mono=1 channel). In the next step, we listened to all recordings and removed test runs (n=14), content-free accidental short recordings (n=29), recordings in which the microphone was covered (n=27), and assessments in which either the recording or the affective state rating was missing (n=24). Moreover, if two consecutive assessments occurred within 15 minutes of each other (n=21), the second assessment was removed unless the audio quality of the first recording was insufficient, in which case the second assessment was kept. Finally, we excluded recordings containing third-party speech (n=8) and recordings with insufficient speech intelligibility due to background noise (n=9). Prior to speech feature extraction, we filtered the remaining 710 recordings usingDeepFilterNet2 [27] to remove background noise.

#### Acoustic Features

We extracted acoustic features using the functionals (version 2) of eGeMAPS [28] from the open-source toolkit openSMILE implemented in Python [24]. Given the limitless number of potential speech features and to increase comparability across studies, this minimalistic set of 88 acoustic features is recommended for use in clinical speech analysis [28]. We added the parameter *speech rate*, which requires transcription of the recordings. We obtained the transcripts using an automatic speech recognition system according to published procedures [29] and corrected the transcripts manually. To determine speech rate, we calculated the ratio of words divided by the duration of the speech sample. In our previous publication [18], we included a subset of three of these speech features (top-down selected: F0semitone From 27.5Hz_sma3nz_stddevNorm, Mean Unvoiced SegmentLength, speech rate) in multilevel model analyses and found an association between each of them and depression severity. In the present work, however, we included all of the described 89 speech features as predictors for depression severity in our ML models.

### ML Analyses

Five ML analyses were conducted to determine the optimal model for predicting ADS-K mean scores from our 89 speech features (Table 1). All analyses used consistent preprocessing, including median imputation for missing data and standard scaling for feature normalization. A variety of models were evaluated: a random chance and a dummy regressor (mean and median; results of the superior are shown) for baseline comparisons, random forest, linear regression, SVR, and XGBoost regression. The models were fine-tuned using nested cross-validation and a systematic grid search to optimize the hyperparameters, ensuring the robustness and reliability of our results using the PHOTON AI (Medical Machine Learning Lab Translational Psychiatry) software package [30].

**Table 1.** Overview of train-test split scenarios.

Train-test split scenario	Explanation	Visualization
Group 5-fold cross-validation	Separation of data points into five bins of approximately equal size, with the condition that each patient’s data are represented in exactly one bin, that is, either in the training set or the test set, but not both. Train on all but one bin, test on the remaining bin. Repetition of the procedure until each bin has been used once as a test bin (5-fold cross-validation).	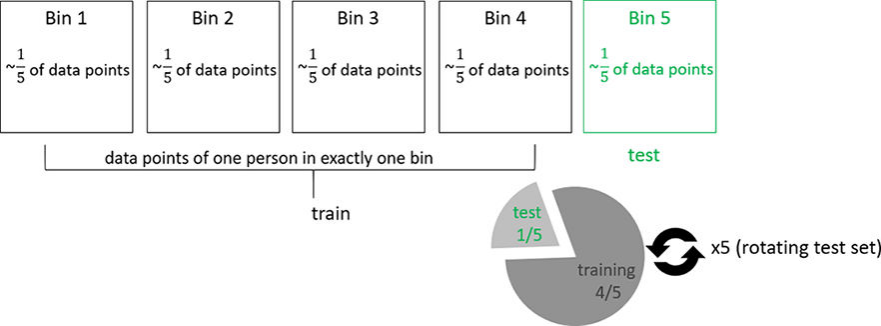 ^a^
Leave-one-subject-out	Train on data from all but one patient. Test on data from the one left-out patient. The procedure was repeated until each subject was used in the test arm (in our study N=22).	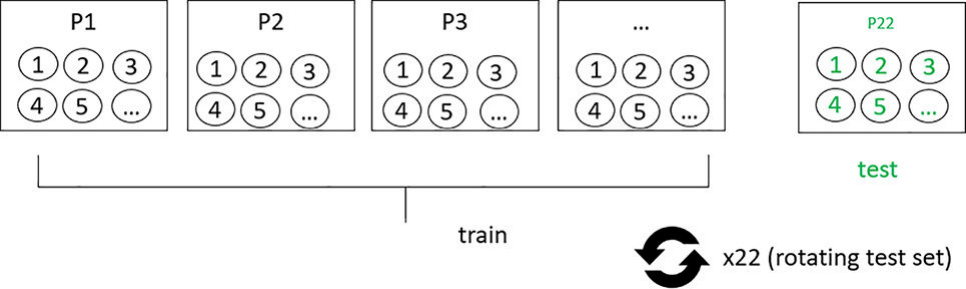
Chronological split	Train on the chronologically first 50% of data, test on the last 50%.	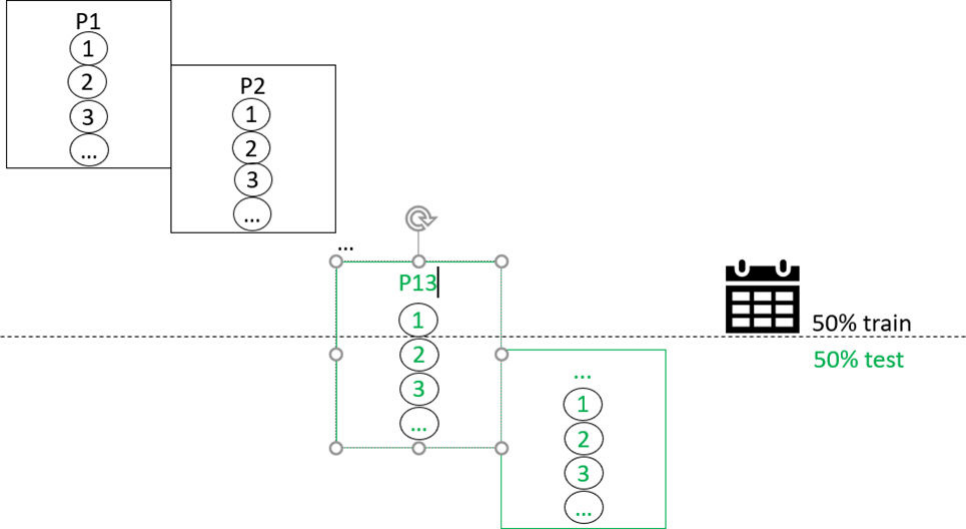
Odd-even split	Odd assessment points were assigned to the training set, even assessment points to the test set. Then the implementation of a 2-fold cross-validation.	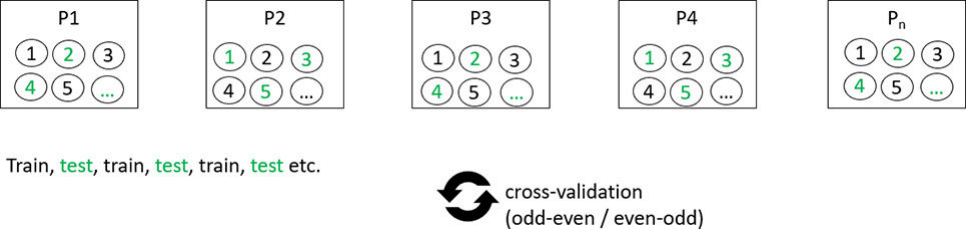
Random split	Data points were randomly assigned to either train or test sets. This was repeated ten times with a 2-fold cross-validation calculated in each repeated run.	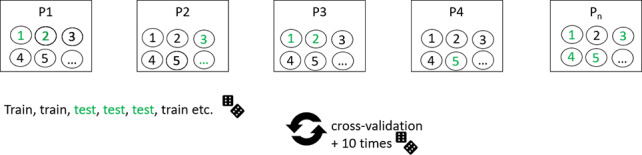

a For visualizations: squares represent data bins in the first row and individual patients in the remaining rows; circles represent individual data points. P: patient.

Model performance was assessed quantitatively using the coefficient of determination (*R*²). This metric evaluates the proportion of variance in the dependent variable that can be explained by the independent variables, providing a clear measure of model effectiveness. It is essential for comparing different regression models in our analysis by quantifying how well each model explains the variability in the dataset. The performance metrics for each model and splitting technique combination were averaged to provide a comprehensive evaluation of model performance.

The calculation of the *R*² score in scikit-learn [31] is executed as follows:


$$R^{2}(y,\hat{y})=1-\frac{\sum_{i=1}^{n}(y_{i}-{\hat{y}}_{i}{)}^{2}}{\sum_{i=1}^{n}(y_{i}-{\bar{y}}_{i}{)}^{2}}$$

where y represents the observed values, $\hat{y}$ represents the predicted values by the model, $\bar{y}$ is the mean of the observed values y, and n is the number of observations.

We also present mean absolute error (MAE) scores which measure how close the predicted and actual values are. MAEs provide a straightforward interpretation given that they are calculated in the same units as the underlying data. Clinical relevance can be inferred.

Higher *R*² scores and lower MAE scores indicate superior model performance. *P* values <.05 are considered to be statistically significant. Negative *R*² scores indicate poor model performance, and in such cases, the *P* value is not of interest.

#### Group 5-Fold Cross-Validation

In our first analytical approach, we used group 5-fold nested cross-validation to assess model performance. Data points were divided into five bins of approximately equal size, ensuring that each patient’s data appeared in only one bin, either in the training set or the test set, but not both. This means that samples from a single patient were treated as a distinct group, ensuring the integrity of individual data within each validation fold. The model was trained on four bins and tested on the remaining bin. The procedure was repeated until each bin had been used as the test bin, completing the 5-fold cross-validation. This approach tested whether the predictive patterns identified could generalize from one group of patients to another by modeling the association between speech features and depression severity across multiple patients.

#### LOSO Split

In the second approach, we used the maximum possible data in a subject-based split for the training set. That is, we used data from all but one patient in the training set with the goal of predicting data from this one unknown patient. This reflects a potential future clinical use case where a trained model is applied to a new, unknown patient. Thus, this analysis tests whether the identified predictive pattern generalizes to an unknown patient.

In the following three approaches, we split the data fifty-fifty by using three different splitting techniques: a chronological split, an odd-even split, and a random split.

#### Chronological Split

In this approach, we used a chronological train-test split where the first 50% of the data (355 data points), ordered by assessment date, were used as the training set and the last 50% were used as the test set (355 data points). Note that our patients were recruited over a time period of 3 years and 2 months. This means that sometimes data were collected from only 1 patient and sometimes from 2 patients at the same time. Specifically, 13 patients of our final sample were enrolled consecutively. For 9 consecutive patients (ie, 9 pairs of patients), there is an overlap in assessment time when comparing the first assessment and the last assessment of an individual patient. Consequently, “earlier” patients are included in the training set, “later” patients only in the test set, and 3 patients in both. No cross-validation was applied, as this would indicate a prediction backward in time. This approach aimed to simulate a realistic prediction scenario by training the models on earlier assessments and testing their performance on later data points, thereby evaluating the predictive performance for future depression severity based on past assessments.

#### Odd-Even Split

This method used a nested 2-fold cross-validation approach, in which patient-wise chronologically sorted data were alternately assigned to the training or test set based on odd and even collection points. As a result, half of the data from each patient is represented in the training set and half in the test set. Importantly, with this splitting mechanism, we assume that both the test and training sets are likely to contain data points from different states, namely severely depressed states and euthymic states right after the intervention. This approach has the advantage that the model is trained with both individual data from depressive and euthymic states, and it avoids having all depressive data in the training set but euthymic data only in the test set. Accordingly, this allows us to model and evaluate the predictive performance of speech features in clinical use cases. For example, predicting the severity of depression in a new depressive episode of a patient with a history of recurrent depression, who is already known by the model.

#### Random Split

Since there is only one way to split data into training and test sets in the odd-even split, we aimed to test the replicability of these findings here. We randomly split our data into test and training sets and performed 2-fold cross-validation. There are 710 *choose* 355=1.612 × 10^143^ ways to randomly split the data into 2 halves. With this splitting mechanism, it is possible that some data points never appear in the training set. Therefore, we repeated this random split ten times and report the mean values.

## Results

### Descriptive Results

Our final dataset consisted of 710 pairs of self-reported depressive momentary states and speech features extracted from concomitantly recorded selfie videos. Self-reported depression severity, as indicated by ADS-K responses (scale 0‐3), was on average 1.2 (SD 0.6). The intraclass correlation coefficient for the ADS-K was 0.47, indicating that 53% of the variance in momentary depression symptoms is attributable to within-person variability. The reliability index of the ADS-K in this study was excellent as evaluated according to McDonald ω (0.87 within-person and 0.90 between-person). Histograms and correlation plots to illustrate our data structure are found in the Multimedia Appendix 2.

### ML Results

#### Overview

We present the performance of each of our 30 ML approaches in Table 2. All combinations of our 6 models (from top to bottom: random chance, dummy regression, random forest regression, linear regression, SVR, and XGBoost regression) and our 5 splitting mechanisms (from left to right: group 5-fold cross-validation, LOSO, chronological split, odd-even split, and random split) are included in the table. We show *R*² scores and MAE along with their *P* values.

**Table 2.** Model performances.

Model	Splitting techniques									
	Group 5-fold cross-validation		Leave-one-subject-out		Chronological split		Odd-even-split		Random split	
	*R*² score	MAE	*R*² score	MAE	*R*² score	MAE	*R*² score	MAE	*R*² score	MAE
Random chance (*P* value)	–3.306 (N/A)^a^	0.920 (N/A)	–6.833 (N/A)	0.910 (N/A)	–2.364 (N/A)	0.941 (N/A)	–2.115 (N/A)	0.920(N/A)	–2.205 (N/A)	0.890(N/A)
Dummy regression (median)	–0.289 (.79)	0.499 (.41)	–3.624 (.92)	0.557 (.72)	–0.107 (.99)	0.491 (.99)	–0.001 (.35)	0.482 (.48)	–0.007 (.72)	0.488 (.84)
Random forest regression (*P* value)	–0.102 (.09)	0.455 (.04)	–4.392 (.99)	0.540 (.29)	–0.213 (.65)	0.519 (.81)	0.336 (<.001)	0.381 (<.001)	0.305 (<.001)	0.396 (<.001)
Linear regression (*P* value)	–25.508 (.67)	0.588 (.50)	–37.258 (.71)	0.602 (.31)	–0.364 (.15)	0.534 (.18)	–0.179 (<.001)	0.445 (<.001)	–0.558 (.06)	0.459 (<.001)
Support vector regression (*P* value)	–0.136 (.008)	0.468 (.004)	–4.006 (.88)	0.570 (.89)	–0.106 (.59)	0.439 (.87)	0.313 (<.001)	0.388 (<.001)	0.293 (<.001)	0.401 (<.001)
XGBoost regression (*P* value)	–0.093 (.07)	0.455 (.03)	–3.568 (.41)	0.550 (.03)	0.084 (.98)	0.442 (.99)	0.339 (<.001)	0.380 (<.001)	0.289 (<.001)	0.399 (<.001)

a N/A: not available.

#### Group 5-Fold Cross-Validation

In our initial analysis using group 5-fold cross-validation, all tested regressors yielded negative *R*^2^ scores and failed to reach a performance above chance level (Table 2). This indicates that none of the models were able to significantly explain the variance of the target variable and thus failed to provide reliable predictive insights for the ADS-K mean scores in this specific setup. The models were not suitable for the dataset under the group 5-fold cross-validation scheme. This finding necessitates a reconsideration of the model parameters, feature selection, or possibly the experimental design to improve predictive performance.

#### LOSO Results

The LOSO approach yielded comparable results. All models tested yielded nonsignificant negative *R*^2^ scores (Table 2). This indicates that none of the models effectively explained the variance of the target variable and all models were unable to predict the mean of the ADS-K scores for an unknown patient in this particular setup.

#### Chronological Split

In the chronological split analysis, none of the models achieved statistically significant results (Table 2). These results suggest that none of the models evaluated were effective in explaining the variance in the ADS-K mean scores or providing reliable predictions in this setup.

#### Odd-Even Split

Overall, the performance of three models tested was above chance level (Table 2). The XGBoost regression emerged as the superior performer, achieving an *R*² score of 0.339 and an MAE of 0.38 (both *P*<.001). These results indicate that approximately 33.9% of the variance in the ADS-K mean scores can be explained by the speech features using this model. The MAE indicates that the mean difference between the predicted and the actual scores is 0.38 units on the ADS-K depression severity scale ranging from 0 to 3. This substantial improvement in model performance of the superior model in this approach compared to our previous ML approaches demonstrates the potential effectiveness of the XGBoost model when data are alternately assigned to training and test sets based on odd and even collection points. This analysis highlights the importance of including both depressive and euthymic data points from the same individual in both the training and test set. In addition to the XGBoost model, the SVR and random forest regression yielded statistically significant results of a descriptively comparable order of magnitude.

#### Random Split

The random forest regression emerged as the superior performer (Table 2) in the random split. The model achieved an *R*² score of 0.305 and an MAE of 0.396 (both *P*<.001). These results indicate that using this model, approximately 30.5% of the variance in the ADS-K mean scores can be explained by the speech features. The MAE of the random forest model indicates that the mean difference between the predicted and the actual scores is 0.396 units on the ADS-K depression severity scale ranging from 0‐3. In addition to the random forest regression, the SVR and XGBoost regression models reached statistical significance with descriptively comparable performance.

## Discussion

### Principal Findings

The objective of this study was to evaluate if speech-based multiparameter ML models and specific train-test splits would significantly increase the prediction of depression severity ratings compared to previous statistical analyses. Uniquely, we used a longitudinal dataset of patients with MDD undergoing sleep deprivation therapy. This approach allows the observation of treatment onset and relapse within a few days, thereby allowing for a maximum of within-person variance of momentary depressive states in our dataset. The most effective ML model (XGBoost regression with odd-even splitting) explains 33.9% of the variance of the target variable depression severity with an MAE of 0.38. It is noteworthy that this represents a 17-fold increase in predictive power over our previous analyses of this (same) dataset, which revealed an *R*²_Hox_ of 2% [18]. It should be noted that in our previous analysis we focused on a subset of 3 speech features, whereas in this work 89 speech features were included into the models. Furthermore, in our previous work, we used inference statistics in the form of multilevel models; and ML here. The present results suggest that integrating a larger number of speech features and allowing for more complex modeling can significantly improve prediction performance. However, these findings need to be replicated in a different sample.

Moreover, our findings revealed that several models reached statistical significance, but with varying predictive power. In short, models in which both the training and the test set contained data from the same patients were successful in predicting depression severity based on speech features (odd-even split and random split). In contrast, all of our models which were tested on data from patients for whom the model was naïve, failed (chronological split, 5-fold cross-validation, and LOSO). Interestingly, for both the odd-even and the random split, three ML models (random forest, SVR, and XGBoost) achieved statistical significance, with an *R*² and MAE of descriptively comparable size. This suggests that these two approaches perform similarly and it is probably not critical which one is ultimately chosen. However, this conclusion must be taken with caution as we did not test the models against each other as this would require orders of magnitude more computational power than all the analyses combined here.

As noted above, all models trying to predict depression scores only of patients for whom the model was naïve, failed. This finding suggests that the predictive patterns do not appear to generalize across patients. This indicates that ML models need to be fine-tuned to the specific patient about whom predictions are to be made. This is consistent with previous research indicating better predictive performance for personalized models compared to generalized models [20]. It underscores the importance of longitudinal datasets, which are still scarce. Only when multiple data points per patient are available for training purposes, that is, longitudinal data, can prediction reach a sufficient level.

In this context, the heterogeneity of the clinical picture of MDD must also be taken into account. Widely used diagnostic criteria allow for more than 400 possible combinations of symptoms [32,33]. This might explain why there is no one-size-fits-all approach, that is, associations from one patient can be easily transferred to another patient. In future work, it might be interesting to test whether models trained and tested on different patients, but with a similar clinical picture, would perform better. For example, a model trained on patients whose clinical picture is strongly characterized by having low energy might be transferable to patients with similar characteristics, but not to patients with a high degree of hyperarousal.

### Limitations

Although our study demonstrates the potential use of speech features in clinical monitoring, particularly of patients with recurrent MDD, some limitations must be mentioned. First, our sample size is relatively small. However, we believe that a unique strength of our dataset is the inclusion of patients with an acute clinical diagnosis of a depressive episode requiring an inpatient stay (rather than subclinical study participants), and the true within-person design. Additionally, due to our longitudinal intervention design, we do have a relatively high number of data points per patient and a meaningful amount of variance in our target variable. Future studies are needed to test the replicability of our findings. Second, although eGeMAPS is a standardized set of speech features recommended for clinical use cases, it may not capture all relevant speech characteristics associated with depression. Nevertheless, we prefer to use predefined feature sets suggested by the community rather than creating our own features to increase the comparability across studies. In light of the previous two arguments, pooling of datasets will become very important in the future, another argument for relying on well-known feature sets. Third, we limited our analyses to 5 different splitting techniques, for each of which we trained over 500 ML models. Nowadays, computational power would allow us to run huge amounts of ML models [34]. However, even with our small set of ML variants, we were still able to demonstrate the importance of individualized ML models with well-designed splitting mechanisms.

### Future Directions

Although we did not test personalized ML models per se in this work, our results support the idea that personalized state-of-the-art approaches, that is, individual ML models, are the most promising [19,35]. A prerequisite for this is the collection of sufficient data points per person in a first step. Importantly, there must be sufficient within-person variance in illness states during this so-called burn-in phase [36]. Once a sufficient amount of data of this patient is available, a first model could be trained. As new data is coming in permanently, the model can be constantly updated with the individual’s data, thus continuously improving its performance. Another idea is to start with a generalized or semipersonalized model (eg, trained on same-sex data) to avoid the cold start problem [36]. Incoming data from the patient could be used to fine-tune the model. This is certainly a complex endeavor that requires patience and perseverance on the part of the patients, but might be worth it once a sufficiently functional model is established. In the long term, this could be particularly helpful for patients with a history of recurrent MDD. To test the feasibility of this, longitudinal studies over even longer time periods than those of the few that already exist are needed.

Moreover, to reduce patient burden, it is even more attractive to use behavioral features that patients do not have to actively collect, such as speech. Since we carry our smartphones with us most of the time anyway, and most people speak naturally in their everyday lives, these features seem promising. However, there are still many ethical and privacy questions with regard to the specific category of speech data. For example, speaker identification algorithms are needed that work reliably, on the fly, and in everyday environments (including varying background noise) to ensure that only the target’s speech is analyzed.

### Conclusion

Our study contributes to the emerging field of digital behavioral markers as indicators of mental health by highlighting the potential and challenges of using speech features to monitor depression. While our results suggest that speech features might be useful in predicting momentary depression severity, future research is needed to evaluate whether these findings can be replicated. Ultimately, speech-based depression monitoring systems could significantly improve patient care in the future.

## Supplementary material

10.2196/64578 Multimedia Appendix 1 ADS-K Items. ADS-K: Allgemeine Depressionsskala.

10.2196/64578 Multimedia Appendix 2 Data visualizations.

## Abbreviations

AA: ambulatory assessment
ADS-K: Allgemeine Depressionsskala
eGeMAPS: Extended Geneva Minimalistic Acoustic Parameter Set
*ICD-10*: *International Statistical Classification of Diseases, Tenth Revision*
LOSO: leave-one-subject-out
MAE: mean absolute error
MDD: major depressive disorder
ML: machine learning
openSMILE: Open-Source Speech and Music Interpretation by Large-Space Extraction
RADAR–CNS: Remote Assessment of Disease and Relapse–Central Nervous System
SVR: support vector regression
XGBoost: Extreme Gradient Boosting

## References

[1] Vos T, Lim SS, Abbafati C (2020). Global burden of 369 diseases and injuries in 204 countries and territories, 1990–2019: a systematic analysis for the Global Burden of Disease Study 2019. The Lancet.

[2] Benasi G, Fava GA, Guidi J (2021). Prodromal symptoms in depression: a systematic review. Psychother Psychosom.

[3] Colombo D, Fernández-Álvarez J, Patané A (2019). Current state and future directions of technology-based ecological momentary assessment and intervention for major depressive disorder: a systematic review. J Clin Med.

[4] Ebrahimi OV, Burger J, Hoffart A, Johnson SU (2021). Within- and across-day patterns of interplay between depressive symptoms and related psychopathological processes: a dynamic network approach during the COVID-19 pandemic. BMC Med.

[5] Fried EI, Flake JK, Robinaugh DJ (2022). Revisiting the theoretical and methodological foundations of depression measurement. Nat Rev Psychol.

[6] Abd-Alrazaq A, AlSaad R, Aziz S (2023). Wearable artificial intelligence for anxiety and depression: scoping review. J Med Internet Res.

[7] Torous J, Kiang MV, Lorme J, Onnela JP (2016). New tools for new research in psychiatry: a scalable and customizable platform to empower data driven smartphone research. JMIR Ment Health.

[8] Ebner-Priemer UW, Mühlbauer E, Neubauer AB (2020). Digital phenotyping: towards replicable findings with comprehensive assessments and integrative models in bipolar disorders. Int J Bipolar Disord.

[9] Trull TJ, Ebner-Priemer U (2014). The role of ambulatory assessment in psychological science. Curr Dir Psychol Sci.

[10] Ebner-Priemer UW, Trull TJ (2009). Ecological momentary assessment of mood disorders and mood dysregulation. Psychol Assess.

[11] Rimti FH, Shahbaz R, Bhatt K, Xiang A (2023). A review of new insights into existing major depressive disorder biomarkers. Heliyon.

[12] De Angel V, Lewis S, White K (2022). Digital health tools for the passive monitoring of depression: a systematic review of methods. NPJ Digit Med.

[13] Zarate D, Stavropoulos V, Ball M, de Sena Collier G, Jacobson NC (2022). Exploring the digital footprint of depression: a PRISMA systematic literature review of the empirical evidence. BMC Psychiatry.

[14] Low DM, Bentley KH, Ghosh SS (2020). Automated assessment of psychiatric disorders using speech: a systematic review. Laryngoscope Investig Otolaryngol.

[15] Kraepelin E, Robertson RM (1921). Manic-Depressive Insanity and Paranoia.

[16] Cummins N, Scherer S, Krajewski J, Schnieder S, Epps J, Quatieri TF (2015). A review of depression and suicide risk assessment using speech analysis. Speech Commun.

[17] Wadle LM, Ebner-Priemer UW (2023). Smart digital phenotyping. Eur Neuropsychopharmacol.

[18] Wadle LM, Ebner-Priemer UW, Foo JC (2024). Speech features as predictors of momentary depression severity in patients with depressive disorder undergoing sleep deprivation therapy: ambulatory assessment pilot study. JMIR Ment Health.

[19] Gerczuk M, Triantafyllopoulos A, Amiriparian S Personalised deep learning for monitoring depressed mood from speech. https://ieeexplore.ieee.org/abstract/document/9991737/.

[20] Campbell EL, Dineley J, Conde P Classifying depression symptom severity: assessment of speech representations in personalized and generalized machine learning models. https://www.isca-archive.org/interspeech_2023/.

[21] Cummins N, Dineley J, Conde P (2023). Multilingual markers of depression in remotely collected speech samples: a preliminary analysis. J Affect Disord.

[22] Wirz-Justice A, Benedetti F (2020). Perspectives in affective disorders: clocks and sleep. Eur J Neurosci.

[23] Matcham F, Barattieri di San Pietro C, Bulgari V (2019). Remote assessment of disease and relapse in major depressive disorder (RADAR-MDD): a multi-centre prospective cohort study protocol. BMC Psychiatry.

[24] Eyben F, Wöllmer M, Schuller B (2010). Opensmile: the munich versatile and fast open-source audio feature extractor.

[25] Montgomery SA, Asberg M (1979). A new depression scale designed to be sensitive to change. Br J Psychiatry.

[26] Hautzinger M (1988). Ein Depressionsmessinstrument für Untersuchungen in der Allgemeinbevölkerung. Diag.

[27] Schröter H, Maier A, Escalante-B AN, Rosenkranz T Deepfilternet2: towards real-time speech enhancement on embedded devices for full-band audio.

[28] Eyben F, Scherer KR, Schuller BW (2016). The Geneva Minimalistic Acoustic Parameter Set (GeMAPS) for voice research and affective computing. IEEE Trans Affect Comput.

[29] Abulimiti A, Weiner J, Schultz T (2020). Automatic speech recognition for ILSE-interviews: longitudinal conversational speech recordings covering aging and cognitive decline. https://www.isca-archive.org/interspeech_2020/.

[30] Leenings R, Winter NR, Plagwitz L (2021). PHOTONAI-A Python API for rapid machine learning model development. PLoS One.

[31] Scikit-learn. https://scikit-learn.org/stable/modules/model_evaluation.html#r2-score.

[32] Goldberg D (2011). The heterogeneity of “major depression.”. World Psychiatry.

[33] Østergaard SD, Jensen SOW, Bech P (2011). The heterogeneity of the depressive syndrome: when numbers get serious. Acta Psychiatr Scand.

[34] Winter NR, Blanke J, Leenings R (2024). A systematic evaluation of machine learning-based biomarkers for major depressive disorder. JAMA Psychiatry.

[35] Wörtwein T, Allen NB, Sheeber LB, Auerbach RP, Cohn JF, Morency LP (2023). Neural mixed effects for nonlinear personalized predictions. https://dl.acm.org/doi/proceedings/10.1145/3577190.

[36] Kathan A, Harrer M, Küster L (2022). Personalised depression forecasting using mobile sensor data and ecological momentary assessment. Front Digit Health.

