# Supplementary material for: Momentary Depression Severity Prediction in Patients With Acute Depression Who Undergo Sleep Deprivation Therapy: Speech-Based Machine Learning Approach
Source: JMIR Ment Health. 2024 Dec 23;11:e64578. doi: 10.2196/64578 (PMC11684135; doi:10.2196/64578)
Supplement: Multimedia Appendix 1 [file mental-v11-e64578-s001.pdf]

Textbox S1. ADS-K items with English translations in italics.

1) Während der letzten Minuten haben mich Dinge beunruhigt, die mir sonst nichts ausmachen.
During the last few minutes, things that normally don't bother me worried me.

2) Während der letzten Minuten konnte ich meine trübsinnige Laune nicht loswerden, obwohl mich meine Freunde / Familie / Mitpatienten versuchten aufzumuntern.
During the last few minutes, I couldn't get rid of my gloomy mood, although my friends / family / fellow patients tried to cheer me up.

3) Während der letzten Minuten hatte ich Mühe mich zu konzentrieren.
During the last few minutes I had trouble concentrating.

4) Während der letzten Minuten war ich deprimiert / niedergeschlagen.
During the last few minutes I was depressed / down.

5) Während der letzten Minuten war alles anstrengend für mich.
During the last minutes everything was exhausting for me.

6) Während der letzten Minuten dachte ich, mein Leben ist ein einziger Fehlschlag.
During the last minutes I thought my life was one big failure.

7) Während der letzten Minuten hatte ich Angst.
During the last minutes I was afraid.

8) Während der letzten Minuten war ich fröhlich gestimmt.
During the last minutes I was in a cheerful mood.

9) Während der letzten Minuten habe ich weniger als sonst geredet.
During the last minutes I talked less than usual.

10) Während der letzten Minuten fühlte ich mich einsam.
During the last minutes I felt lonely.

11) Während der letzten Minuten habe ich das Leben genossen.
During the last minutes I enjoyed life.

12) Während der letzten Minuten war ich traurig.
During the last minutes I felt sad.

13) Während der letzten Minuten hatte ich das Gefühl, dass mich die Leute nicht leiden können.
During the last minutes I felt that people didn't like me.

14) Während der letzten Minuten konnte ich mich zu nichts aufraffen.
During the last minutes I couldn't get myself up to do anything.
