# Supplementary material for: Momentary Depression Severity Prediction in Patients With Acute Depression Who Undergo Sleep Deprivation Therapy: Speech-Based Machine Learning Approach
Source: JMIR Ment Health. 2024 Dec 23;11:e64578. doi: 10.2196/64578 (PMC11684135; doi:10.2196/64578)
Supplement: Multimedia Appendix 2 [file mental-v11-e64578-s002.pdf]

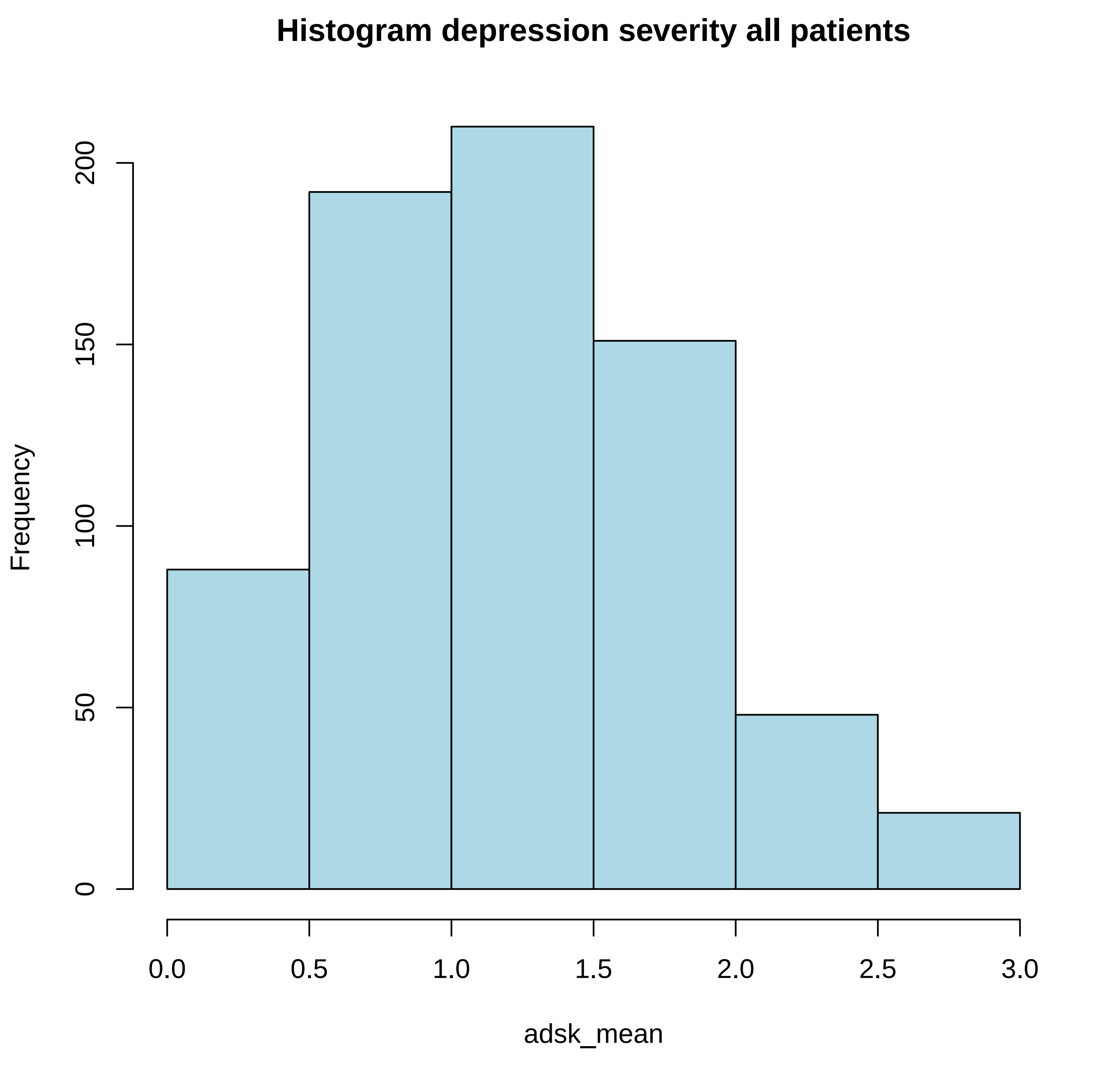
Histogram depression severity all patients
Frequency
0
50
100
150
200
0.0
0.5
1.0
1.5
2.0
2.5
3.0
adsk_mean

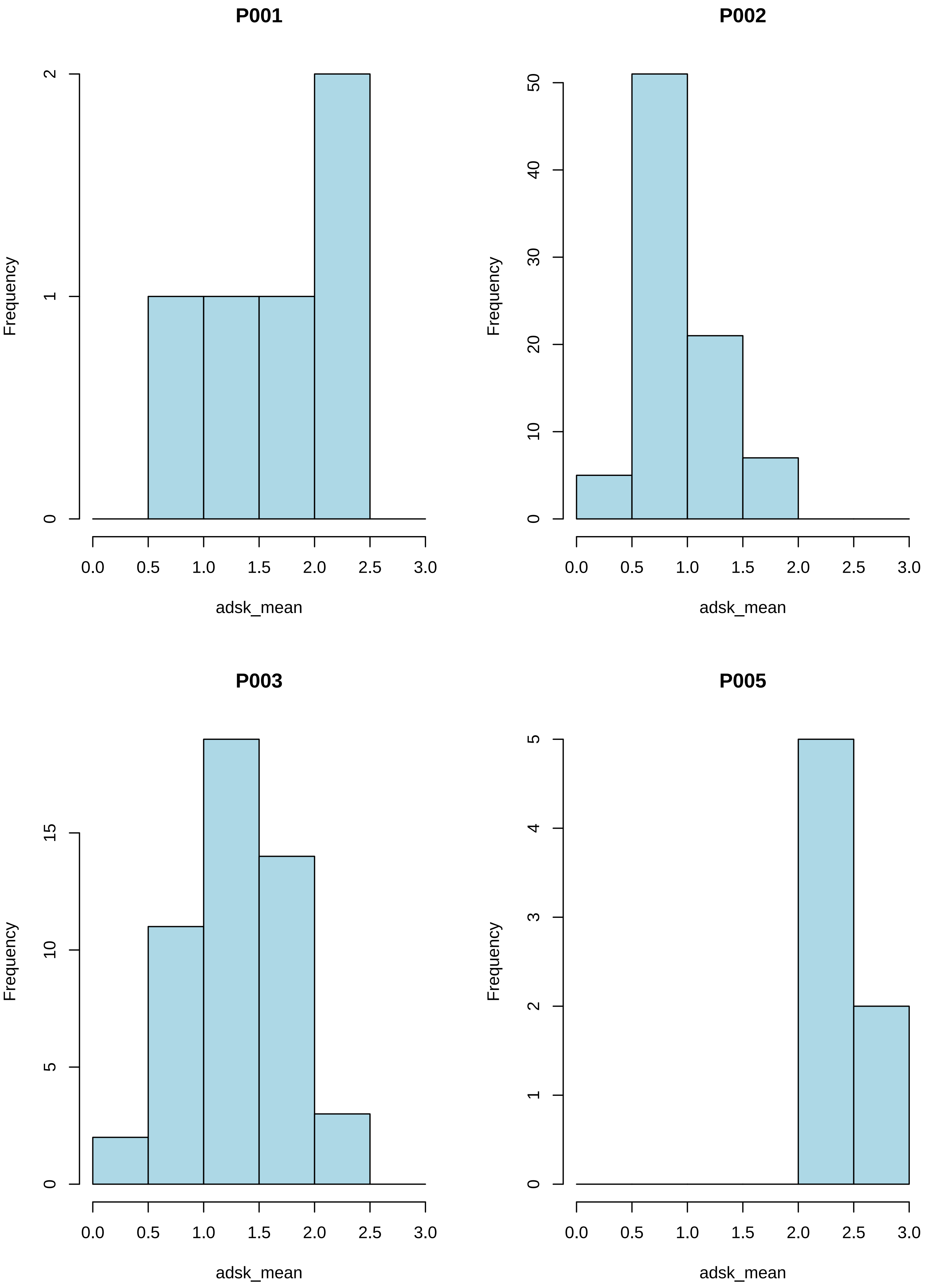
P001
Frequency
0
1
2
0.0
0.5
1.0
1.5
2.0
2.5
3.0
adsk_mean
P002
Frequency
0
10
20
30
40
50
0.0
0.5
1.0
1.5
2.0
2.5
3.0
adsk_mean
P003
Frequency
0
5
10
15
0.0
0.5
1.0
1.5
2.0
2.5
3.0
adsk_mean
P005
Frequency
0
1
2
3
4
5
0.0
0.5
1.0
1.5
2.0
2.5
3.0
adsk_mean

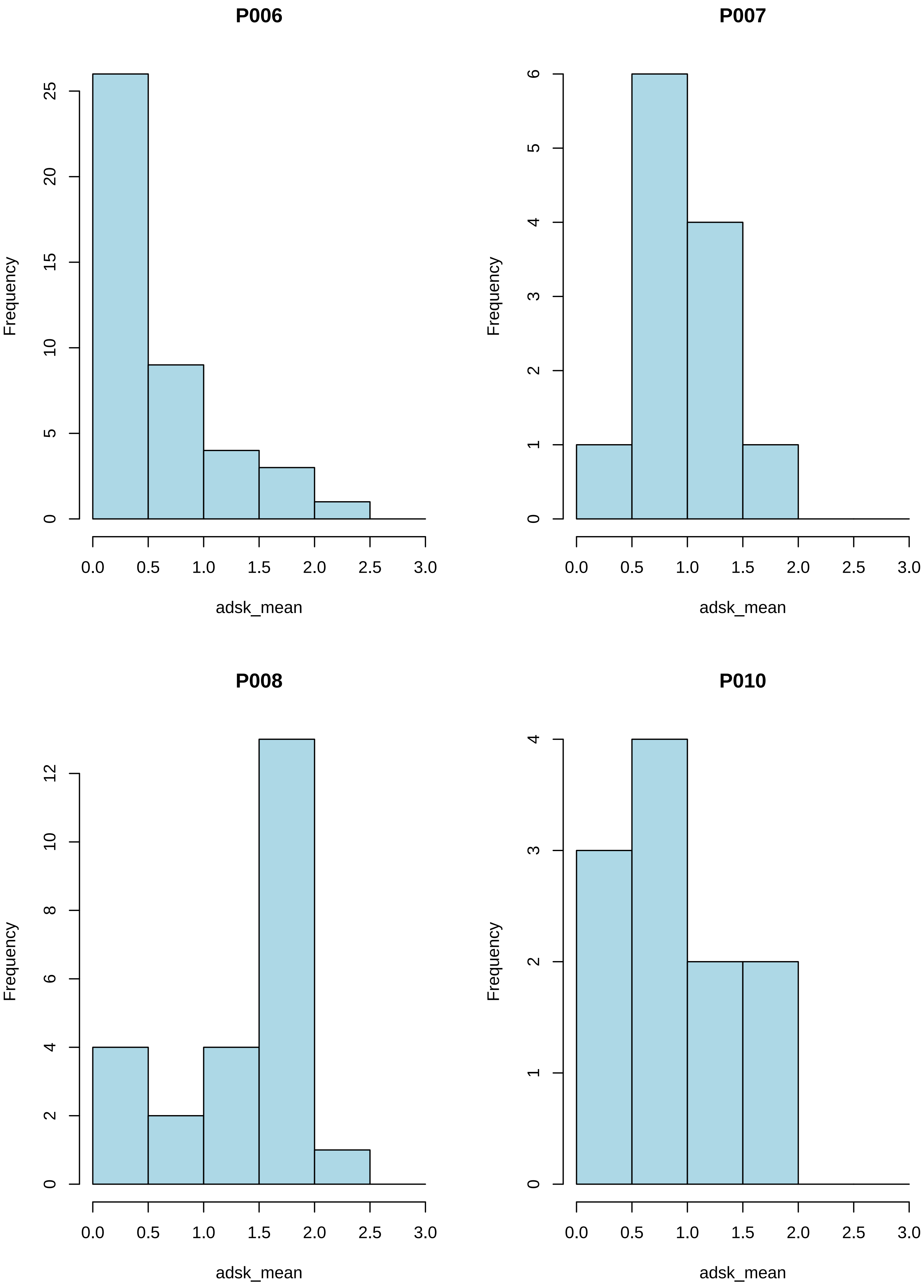

P006
P007
P008
P010
Frequency
adsk_mean
0.0
0.5
1.0
1.5
2.0
2.5
3.0
0
5
10
15
20
25
1
2
3
4
6
8
12

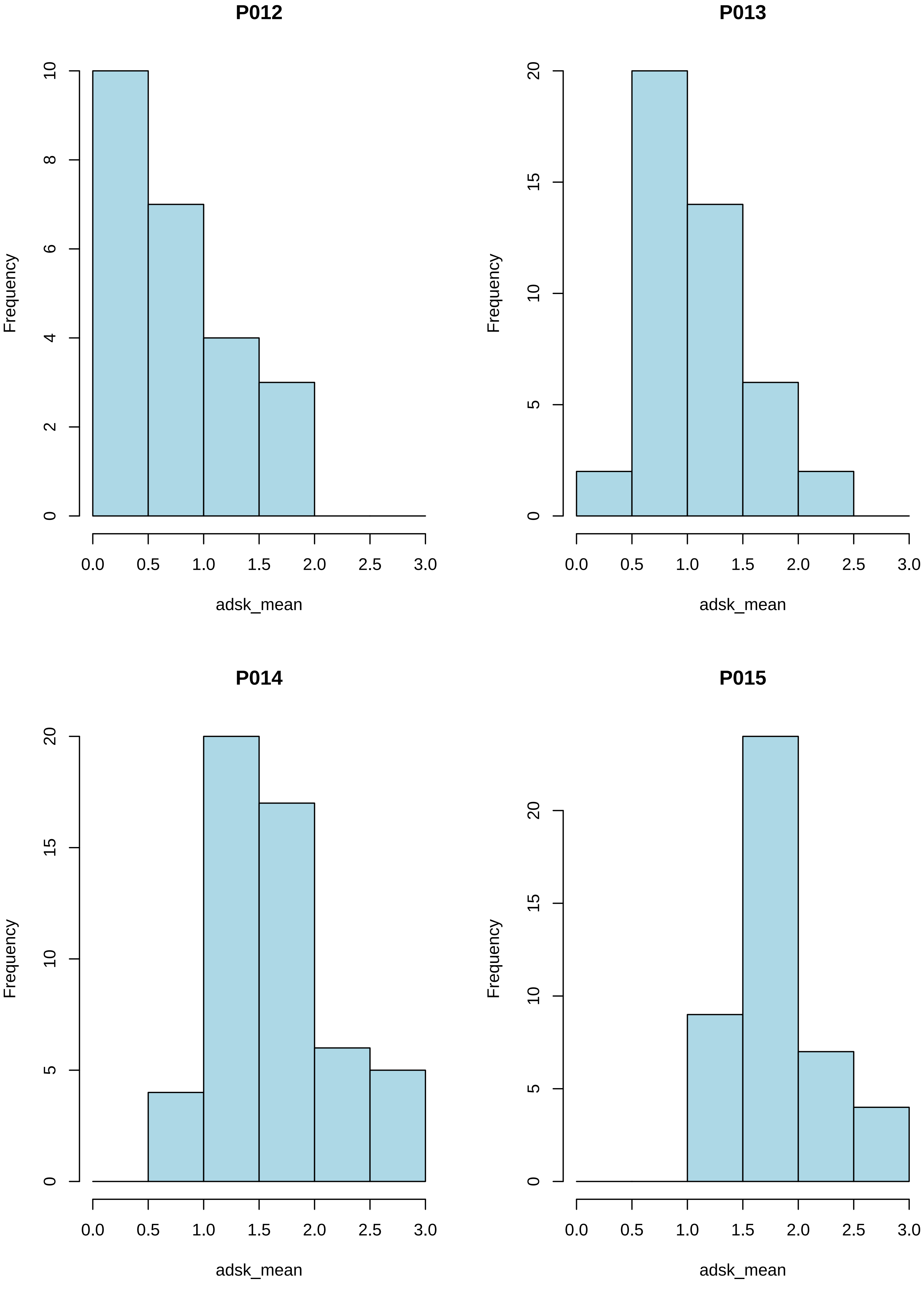
P012
P013
P014
P015
Frequency
adsk_mean
0.0
0.5
1.0
1.5
2.0
2.5
3.0
0
2
4
6
8
10
5
15
20

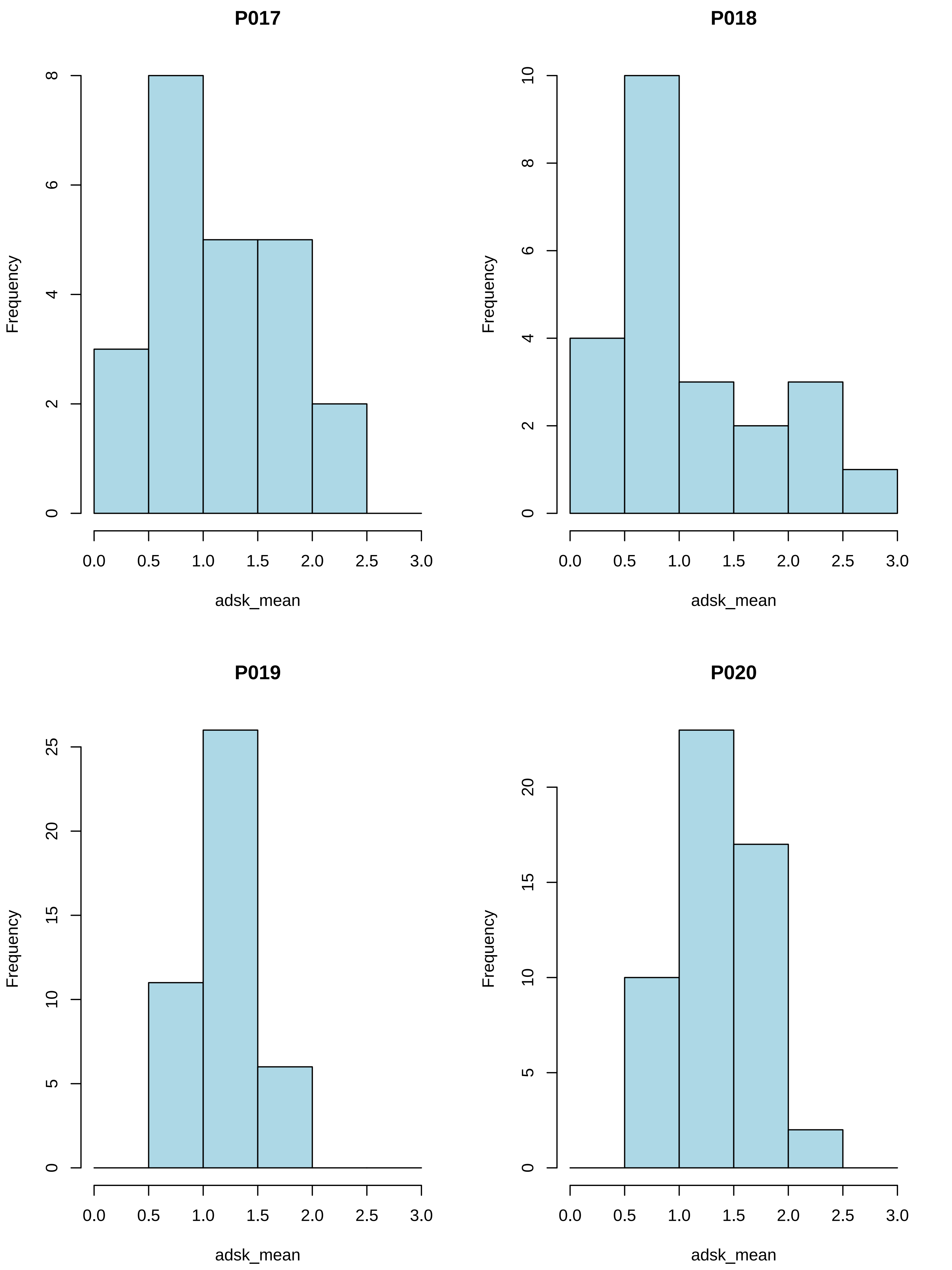
P017
P018
P019
P020
Frequency
adsk_mean
0.0 0.5 1.0 1.5 2.0 2.5 3.0
0 2 4 6 8
0 2 4 6 8 10
0 5 10 15 20 25
0 5 10 15 20

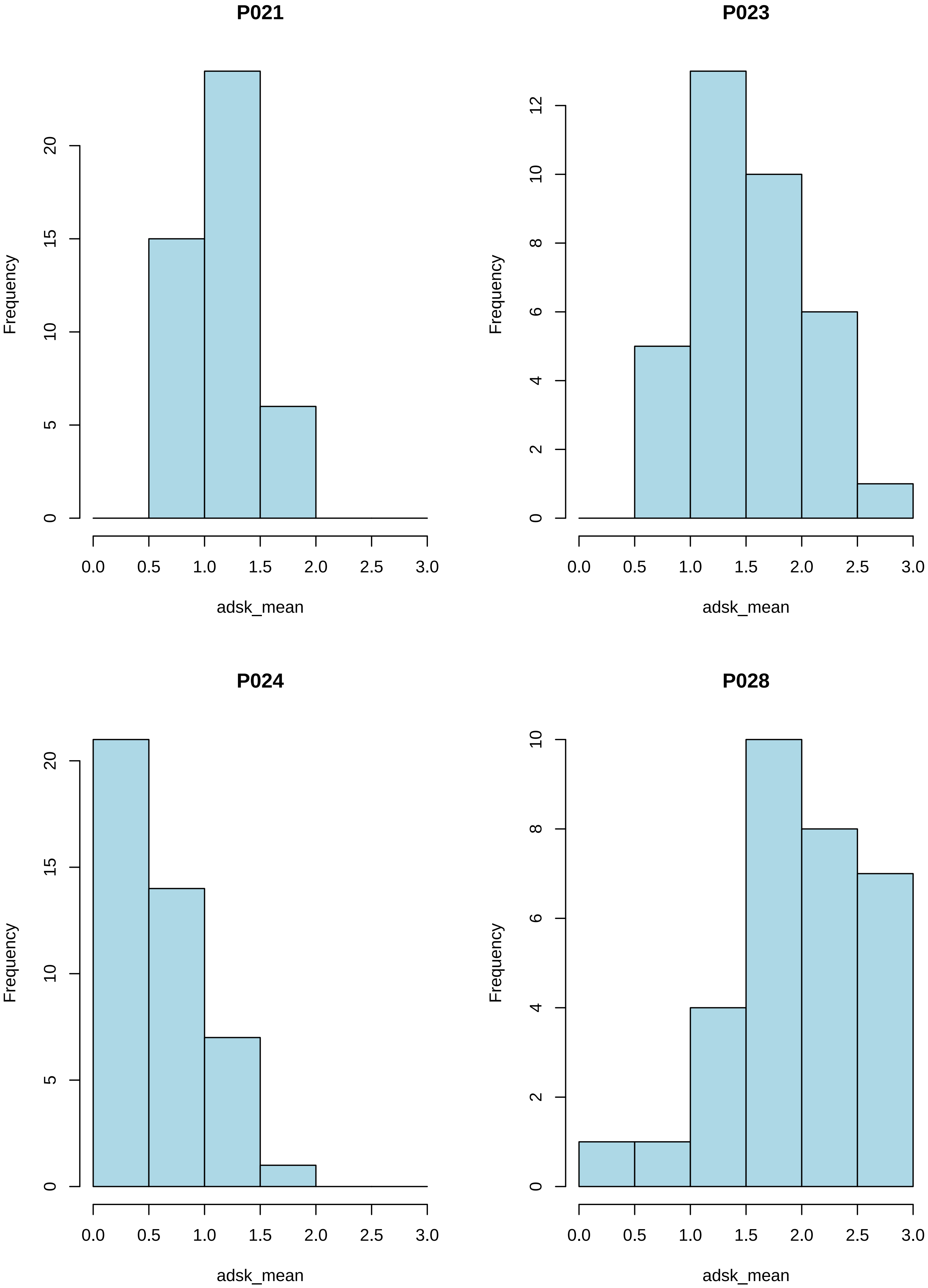
P021
P023
P024
P028
Frequency
adsk_mean
0
5
10
15
20
0.0
0.5
1.0
1.5
2.0
2.5
3.0
2
4
6
8
12

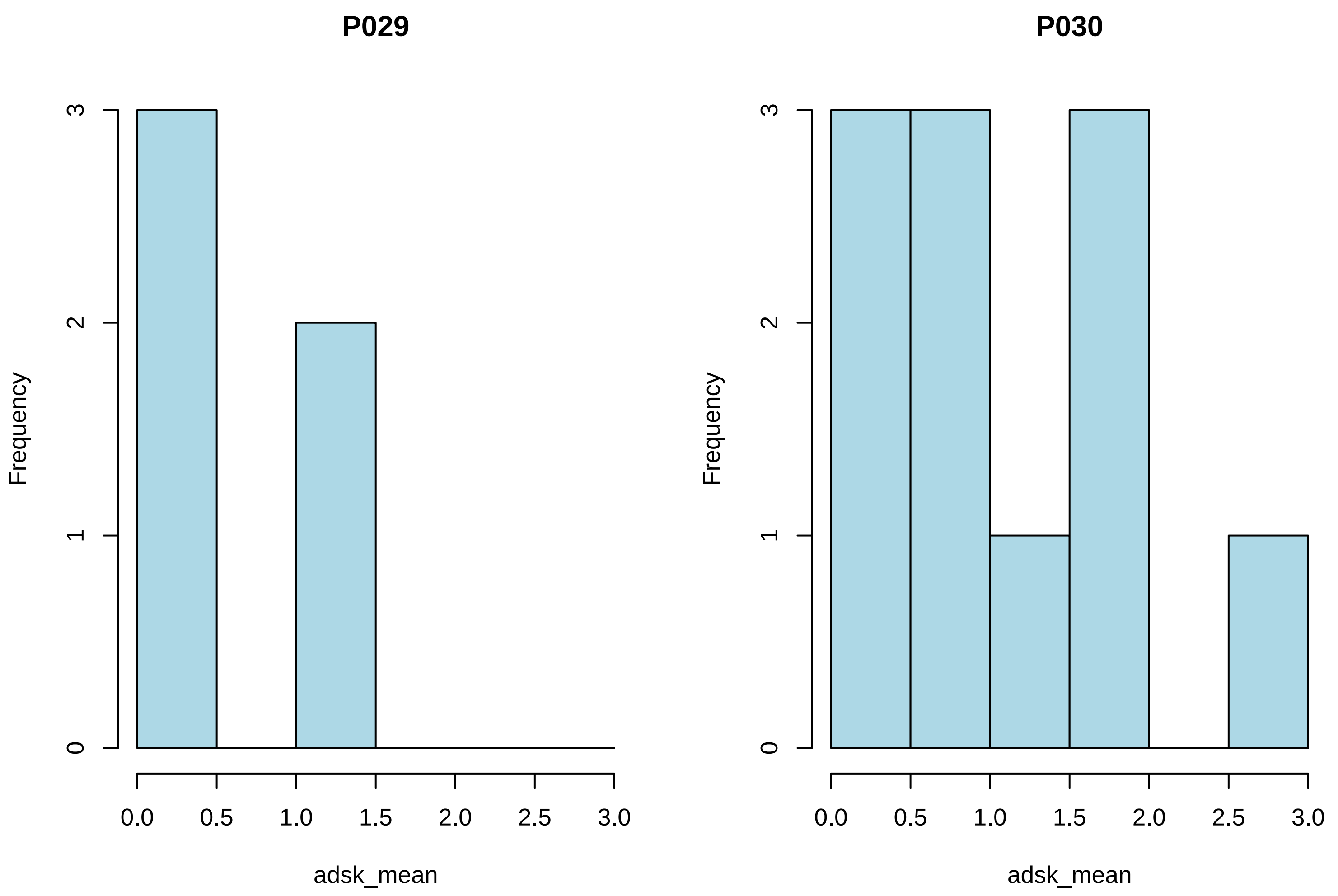
P029
Frequency
3
2
1
0
0.0
0.5
1.0
1.5
2.0
2.5
3.0
adsk_mean
P030
Frequency
3
2
1
0
0.0
0.5
1.0
1.5
2.0
2.5
3.0
adsk_mean

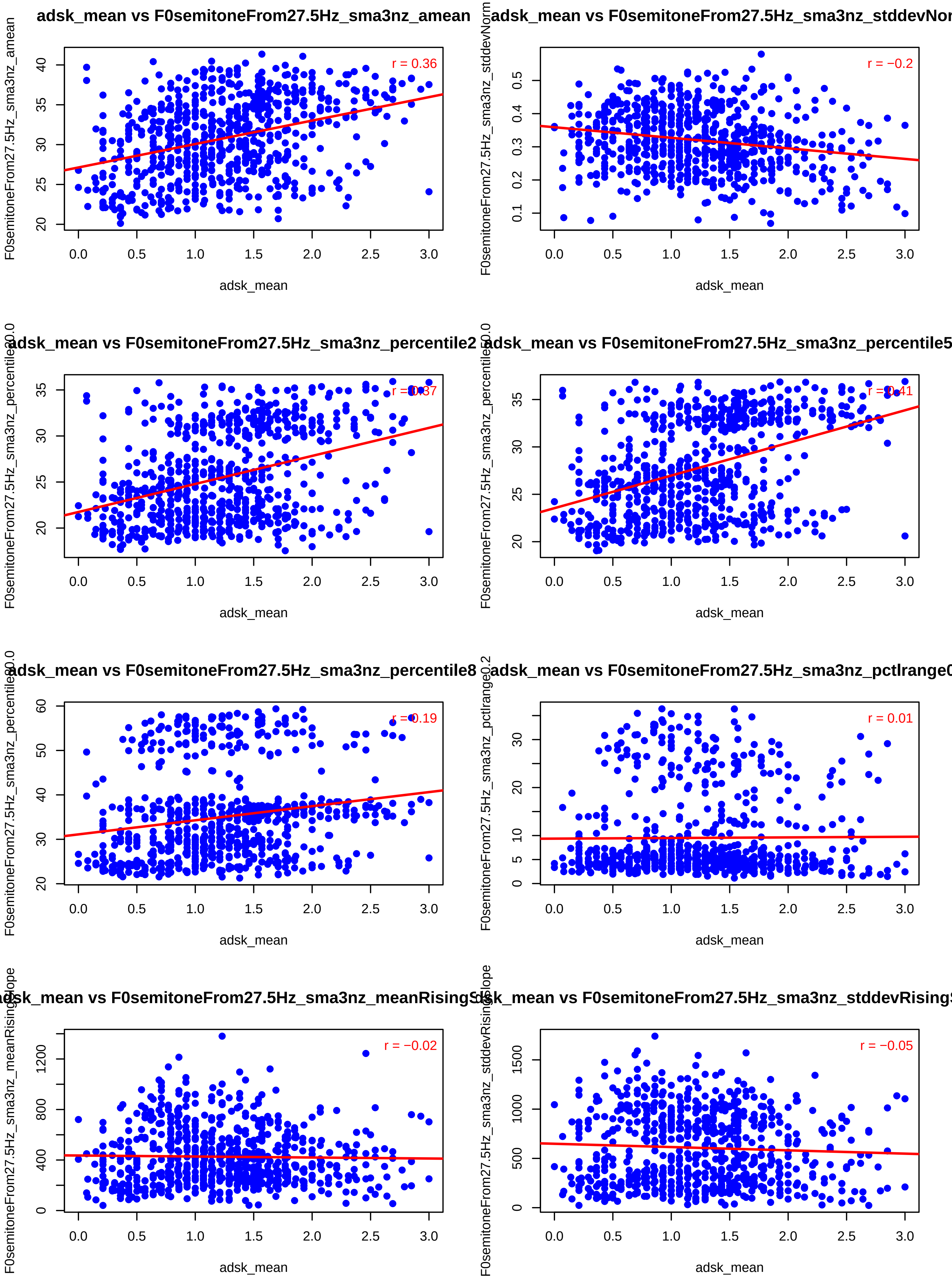
adsk_mean vs F0semitoneFrom27.5Hz_sma3nz_amean
r = 0.36
F0semitoneFrom27.5Hz_sma3nz_amean
adsk_mean
adsk_mean vs F0semitoneFrom27.5Hz_sma3nz_stddevNor
r = −0.2
F0semitoneFrom27.5Hz_sma3nz_stddevNorm
adsk_mean
adsk_mean vs F0semitoneFrom27.5Hz_sma3nz_percentile2
r = 0.37
adsk_mean
adsk_mean vs F0semitoneFrom27.5Hz_sma3nz_percentile5
r = 0.41
adsk_mean
adsk_mean vs F0semitoneFrom27.5Hz_sma3nz_percentile8
r = 0.19
adsk_mean
adsk_mean vs F0semitoneFrom27.5Hz_sma3nz_pctlrange0
r = 0.01
adsk_mean
adsk_mean vs F0semitoneFrom27.5Hz_sma3nz_meanRisingSlope
r = −0.02
adsk_mean
adsk_mean vs F0semitoneFrom27.5Hz_sma3nz_stddevRising
r = −0.05
adsk_mean

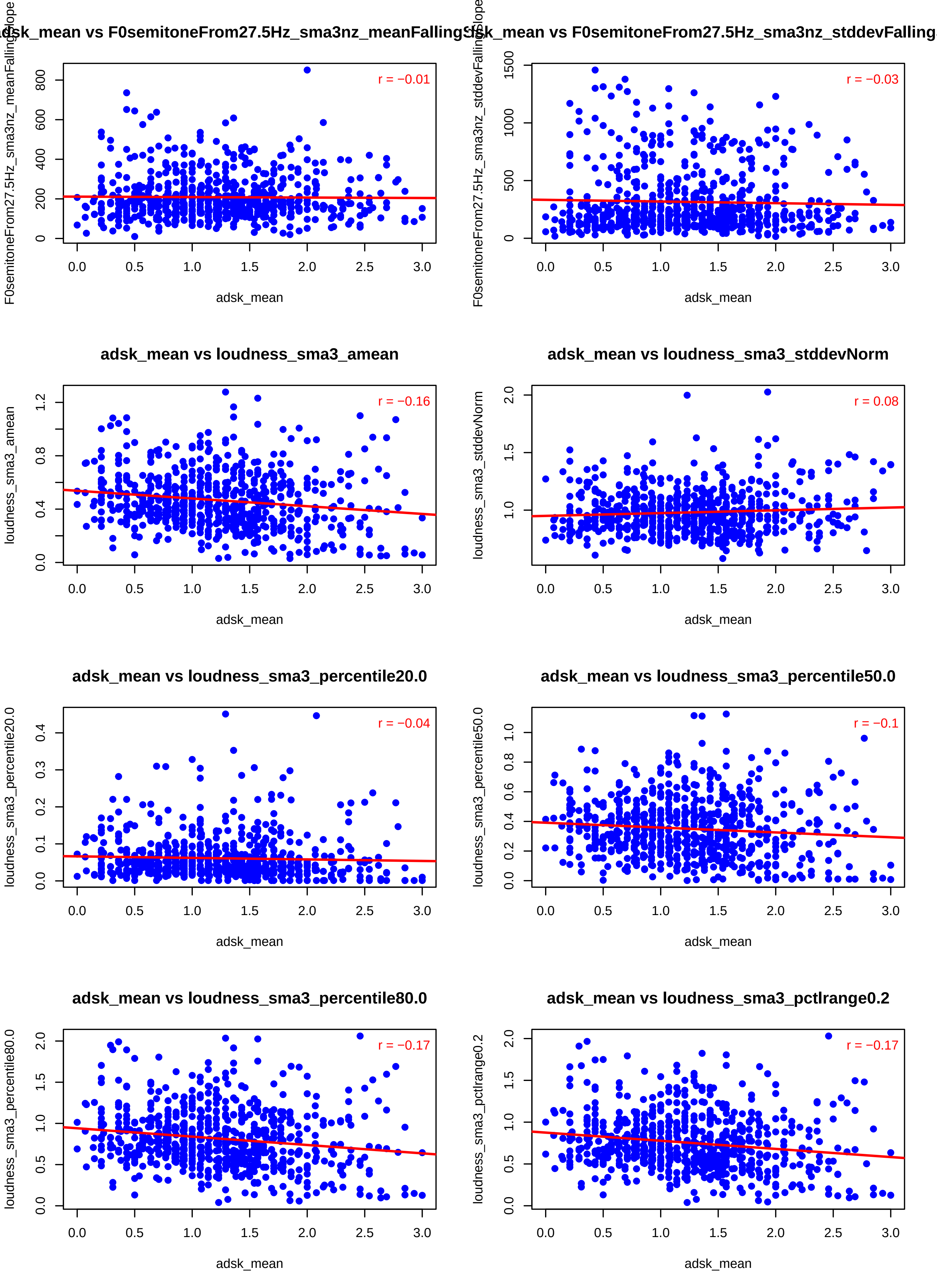

dsk_mean vs F0semitoneFrom27.5Hz_sma3nz_meanFalling
F0semitoneFrom27.5Hz_sma3nz_meanFallingSlope
r = −0.01
adsk_mean
sk_mean vs F0semitoneFrom27.5Hz_sma3nz_stddevFalling
F0semitoneFrom27.5Hz_sma3nz_stddevFallingSlope
r = −0.03
adsk_mean
adsk_mean vs loudness_sma3_amean
loudness_sma3_amean
r = −0.16
adsk_mean
adsk_mean vs loudness_sma3_stddevNorm
loudness_sma3_stddevNorm
r = 0.08
adsk_mean
adsk_mean vs loudness_sma3_percentile20.0
loudness_sma3_percentile20.0
r = −0.04
adsk_mean
adsk_mean vs loudness_sma3_percentile50.0
loudness_sma3_percentile50.0
r = −0.1
adsk_mean
adsk_mean vs loudness_sma3_percentile80.0
loudness_sma3_percentile80.0
r = −0.17
adsk_mean
adsk_mean vs loudness_sma3_pctlrange0.2
loudness_sma3_pctlrange0.2
r = −0.17
adsk_mean

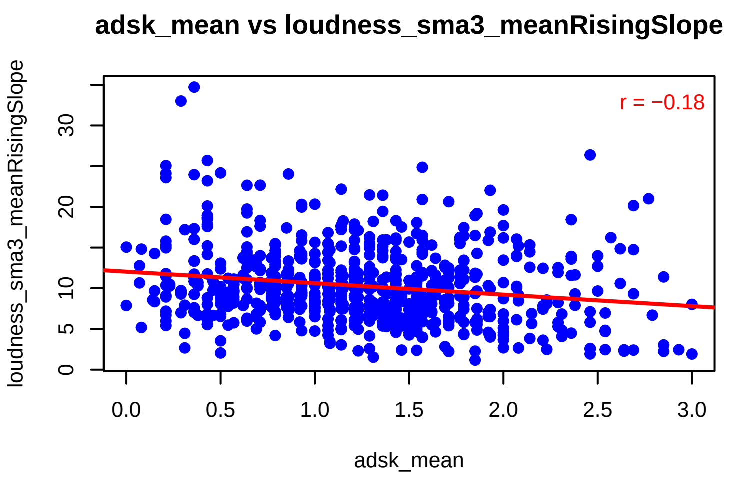
adsk_mean vs loudness_sma3_meanRisingSlope
r = −0.18
loudness_sma3_meanRisingSlope
0
5
10
20
30
0.0
0.5
1.0
1.5
2.0
2.5
3.0
adsk_mean

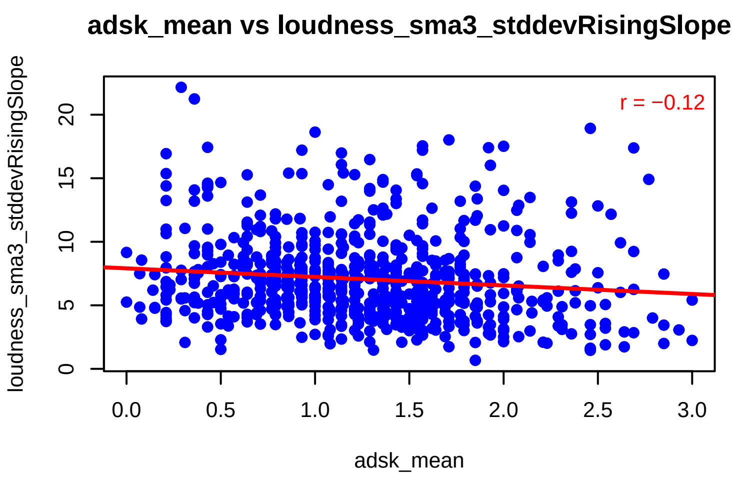
adsk_mean vs loudness_sma3_stddevRisingSlope
r = −0.12
loudness_sma3_stddevRisingSlope
0
5
10
15
20
0.0
0.5
1.0
1.5
2.0
2.5
3.0
adsk_mean

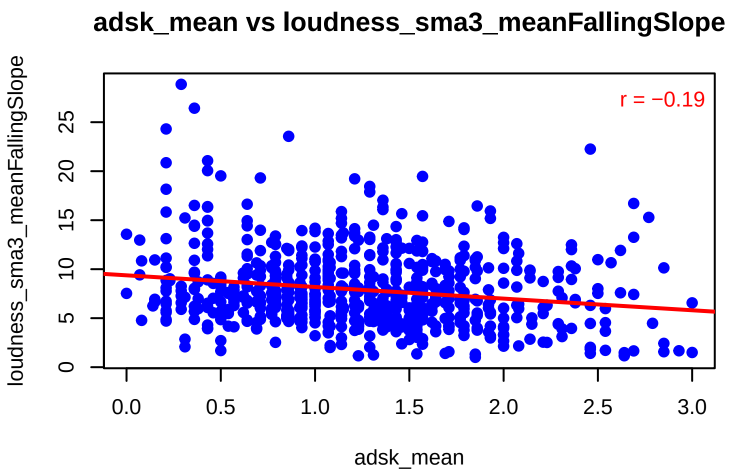
adsk_mean vs loudness_sma3_meanFallingSlope
r = −0.19
loudness_sma3_meanFallingSlope
0
5
10
15
20
25
0.0
0.5
1.0
1.5
2.0
2.5
3.0
adsk_mean

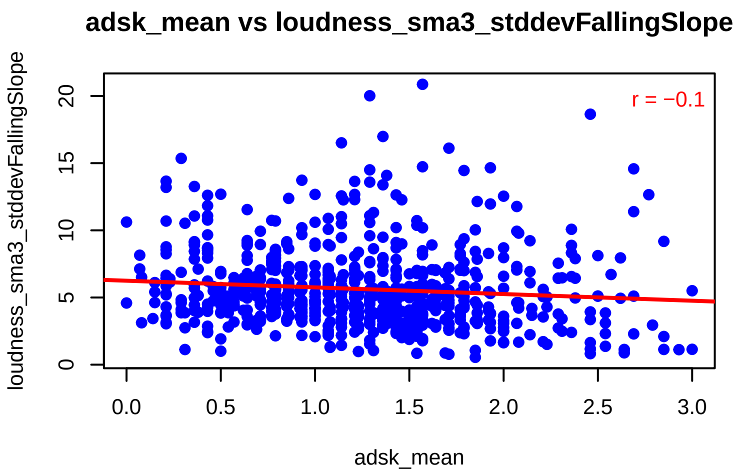
adsk_mean vs loudness_sma3_stddevFallingSlope
r = −0.1
loudness_sma3_stddevFallingSlope
0
5
10
15
20
0.0
0.5
1.0
1.5
2.0
2.5
3.0
adsk_mean

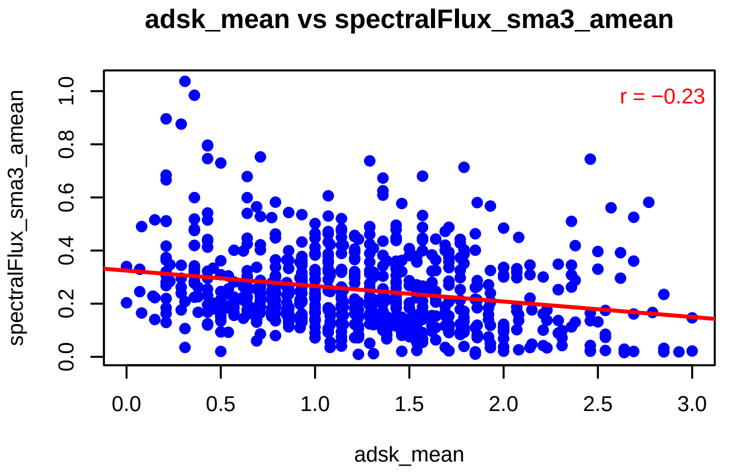
adsk_mean vs spectralFlux_sma3_amean
r = −0.23
spectralFlux_sma3_amean
0.0
0.2
0.4
0.6
0.8
1.0
0.0
0.5
1.0
1.5
2.0
2.5
3.0
adsk_mean

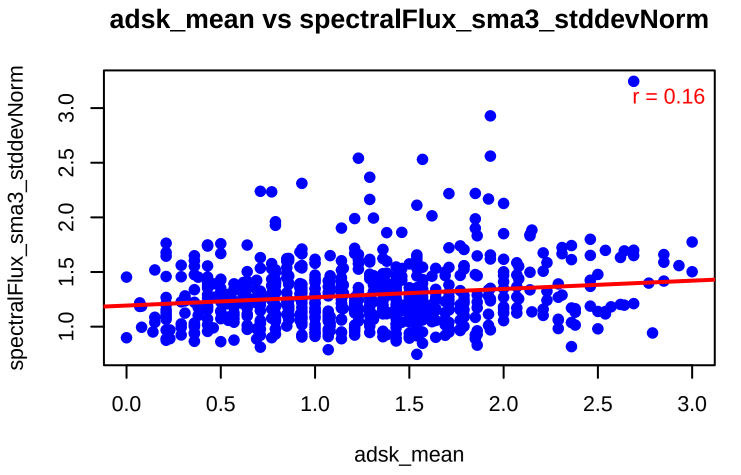
adsk_mean vs spectralFlux_sma3_stddevNorm
r = 0.16
spectralFlux_sma3_stddevNorm
1.0
1.5
2.0
2.5
3.0
0.0
0.5
1.0
1.5
2.0
2.5
3.0
adsk_mean

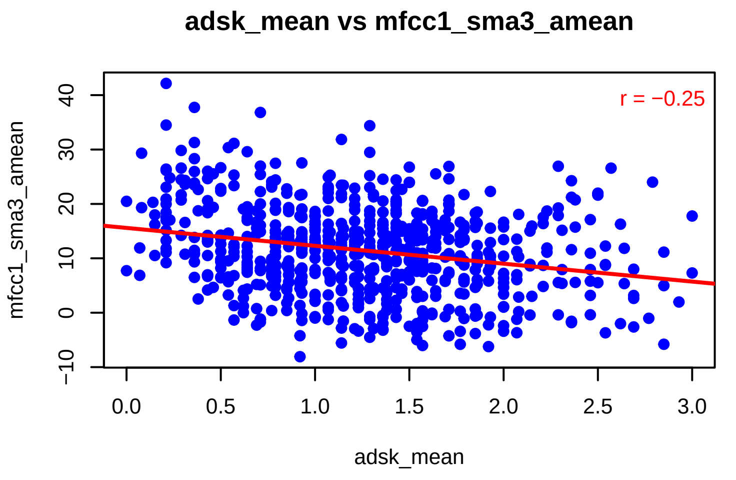
adsk_mean vs mfcc1_sma3_amean
r = −0.25
mfcc1_sma3_amean
−10
0
10
20
30
40
0.0
0.5
1.0
1.5
2.0
2.5
3.0
adsk_mean

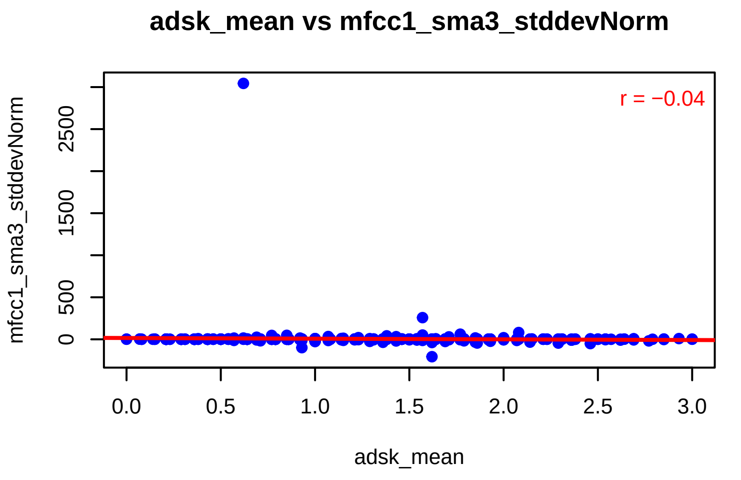
adsk_mean vs mfcc1_sma3_stddevNorm
r = −0.04
mfcc1_sma3_stddevNorm
0
500
1500
2500
0.0
0.5
1.0
1.5
2.0
2.5
3.0
adsk_mean

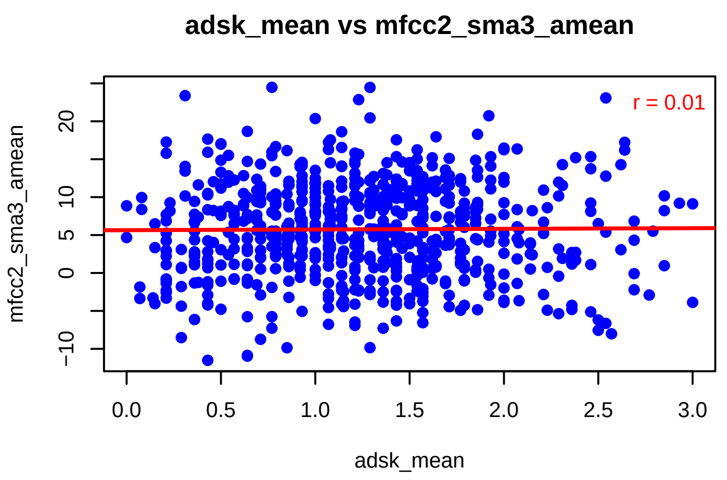
adsk_mean vs mfcc2_sma3_amean
r = 0.01
mfcc2_sma3_amean
adsk_mean

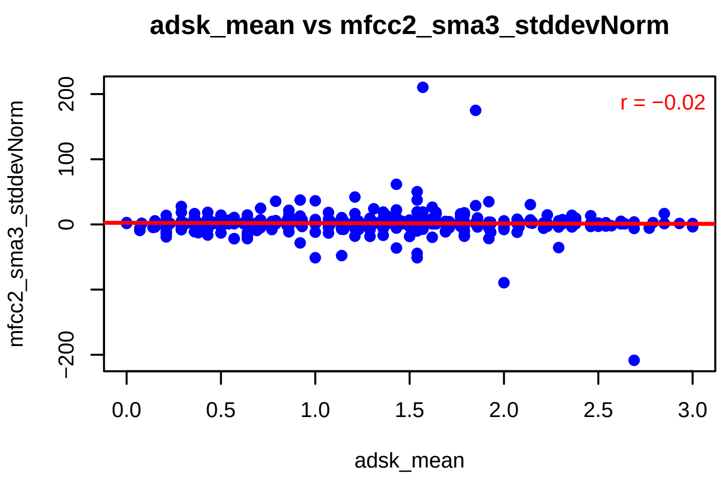
adsk_mean vs mfcc2_sma3_stddevNorm
r = −0.02
mfcc2_sma3_stddevNorm
adsk_mean

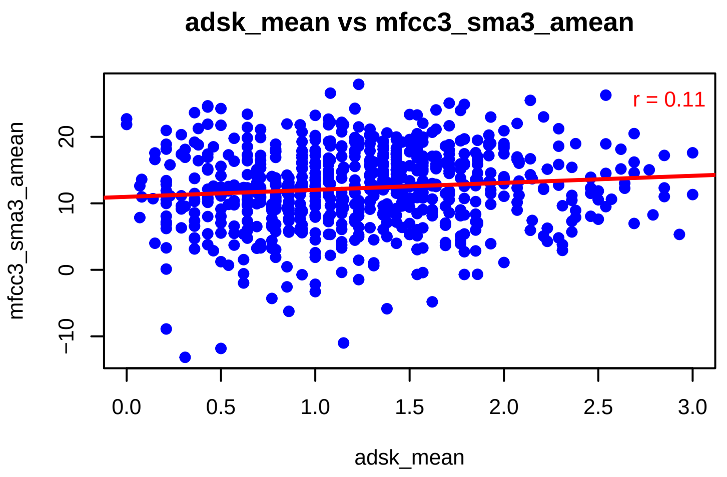
adsk_mean vs mfcc3_sma3_amean
r = 0.11
mfcc3_sma3_amean
adsk_mean

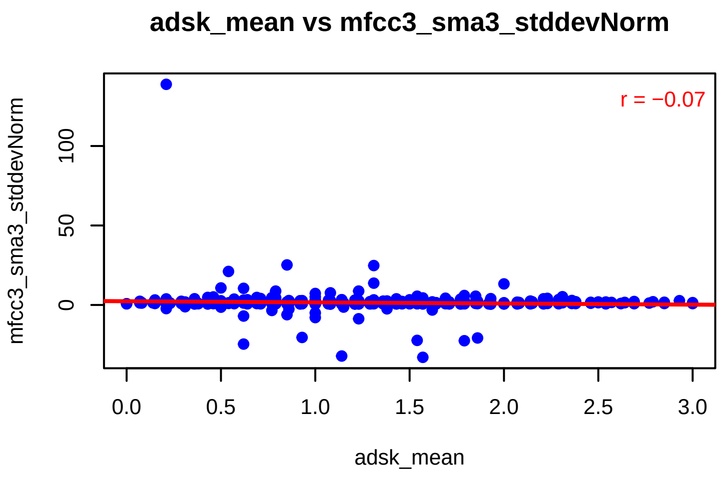
adsk_mean vs mfcc3_sma3_stddevNorm
r = −0.07
mfcc3_sma3_stddevNorm
adsk_mean

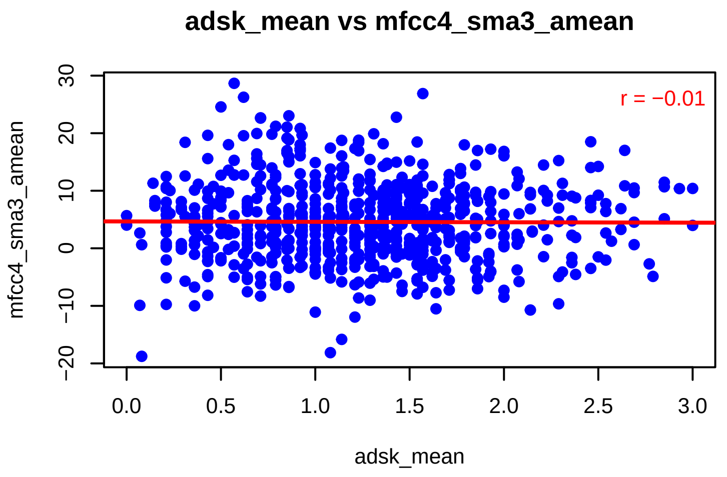
adsk_mean vs mfcc4_sma3_amean
r = −0.01
mfcc4_sma3_amean
adsk_mean

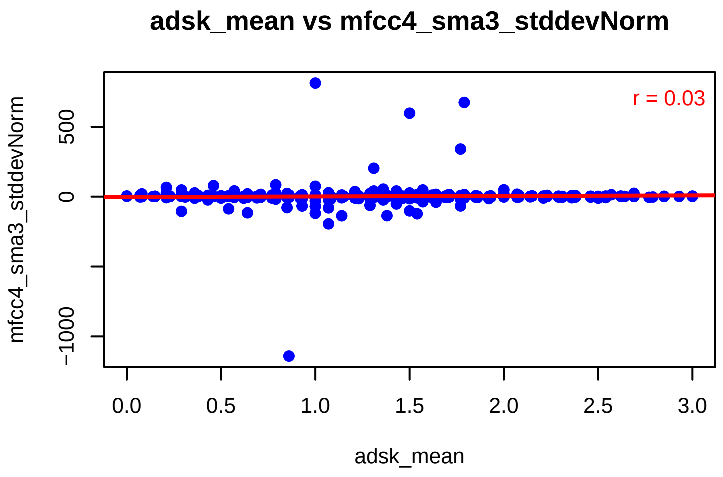
adsk_mean vs mfcc4_sma3_stddevNorm
r = 0.03
mfcc4_sma3_stddevNorm
adsk_mean

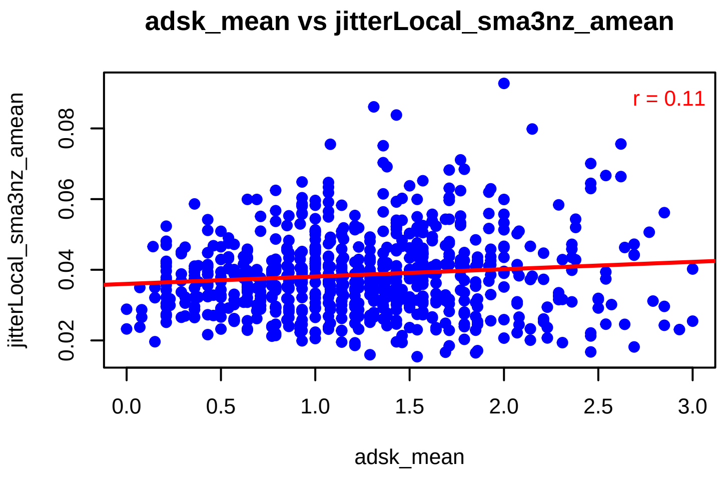
adsk_mean vs jitterLocal_sma3nz_amean
r = 0.11
jitterLocal_sma3nz_amean
adsk_mean

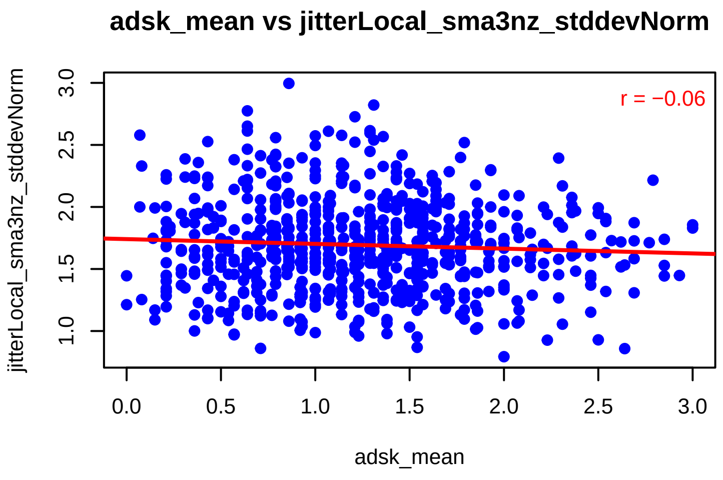
adsk_mean vs jitterLocal_sma3nz_stddevNorm
r = −0.06
jitterLocal_sma3nz_stddevNorm
adsk_mean

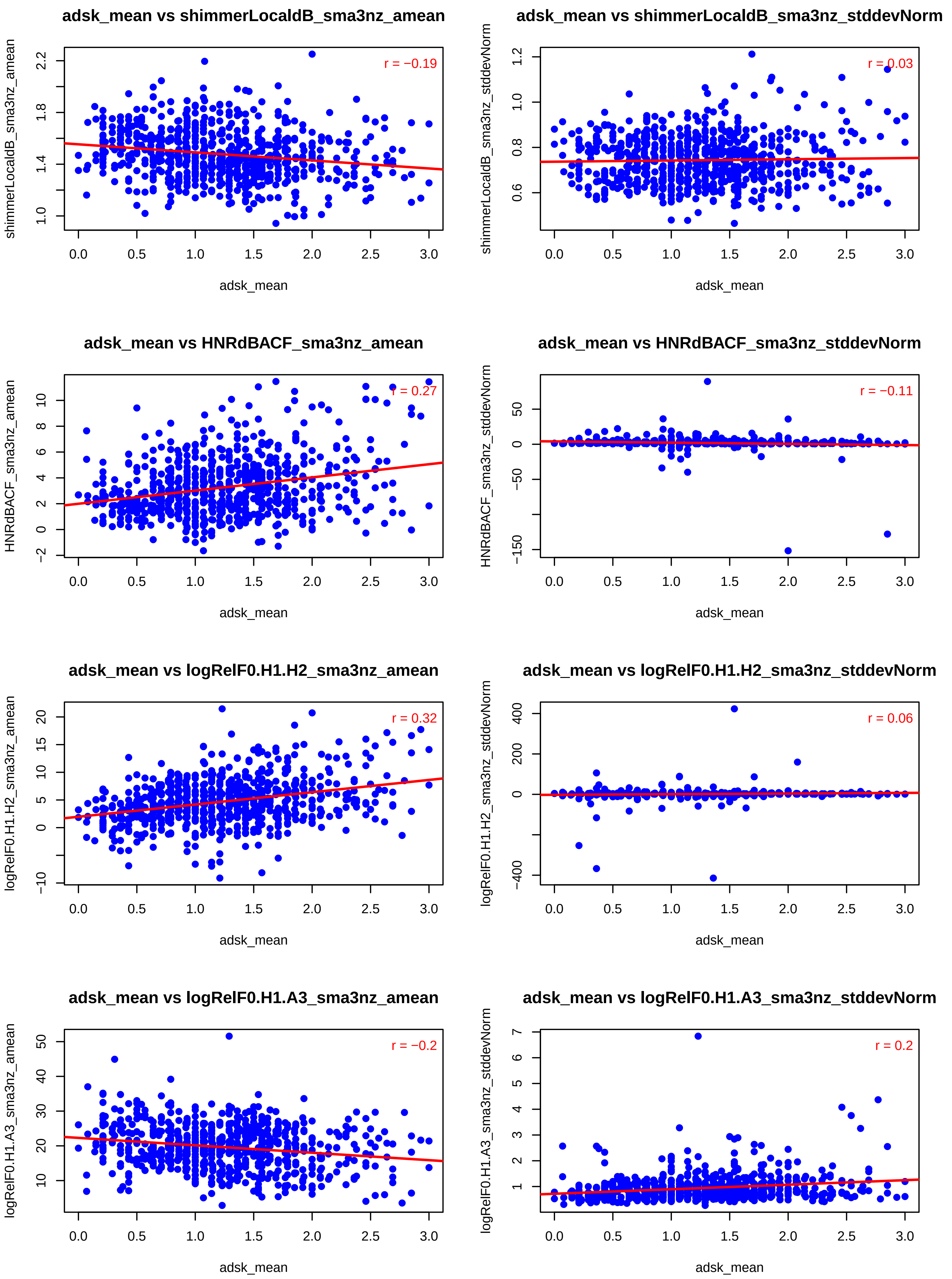
adsk_mean vs shimmerLocaldB_sma3nz_amean
r = −0.19
shimmerLocaldB_sma3nz_amean
adsk_mean
adsk_mean vs shimmerLocaldB_sma3nz_stddevNorm
r = 0.03
shimmerLocaldB_sma3nz_stddevNorm
adsk_mean
adsk_mean vs HNRdBACF_sma3nz_amean
r = 0.27
HNRdBACF_sma3nz_amean
adsk_mean
adsk_mean vs HNRdBACF_sma3nz_stddevNorm
r = −0.11
HNRdBACF_sma3nz_stddevNorm
adsk_mean
adsk_mean vs logRelF0.H1.H2_sma3nz_amean
r = 0.32
logRelF0.H1.H2_sma3nz_amean
adsk_mean
adsk_mean vs logRelF0.H1.H2_sma3nz_stddevNorm
r = 0.06
logRelF0.H1.H2_sma3nz_stddevNorm
adsk_mean
adsk_mean vs logRelF0.H1.A3_sma3nz_amean
r = −0.2
logRelF0.H1.A3_sma3nz_amean
adsk_mean
adsk_mean vs logRelF0.H1.A3_sma3nz_stddevNorm
r = 0.2
logRelF0.H1.A3_sma3nz_stddevNorm
adsk_mean

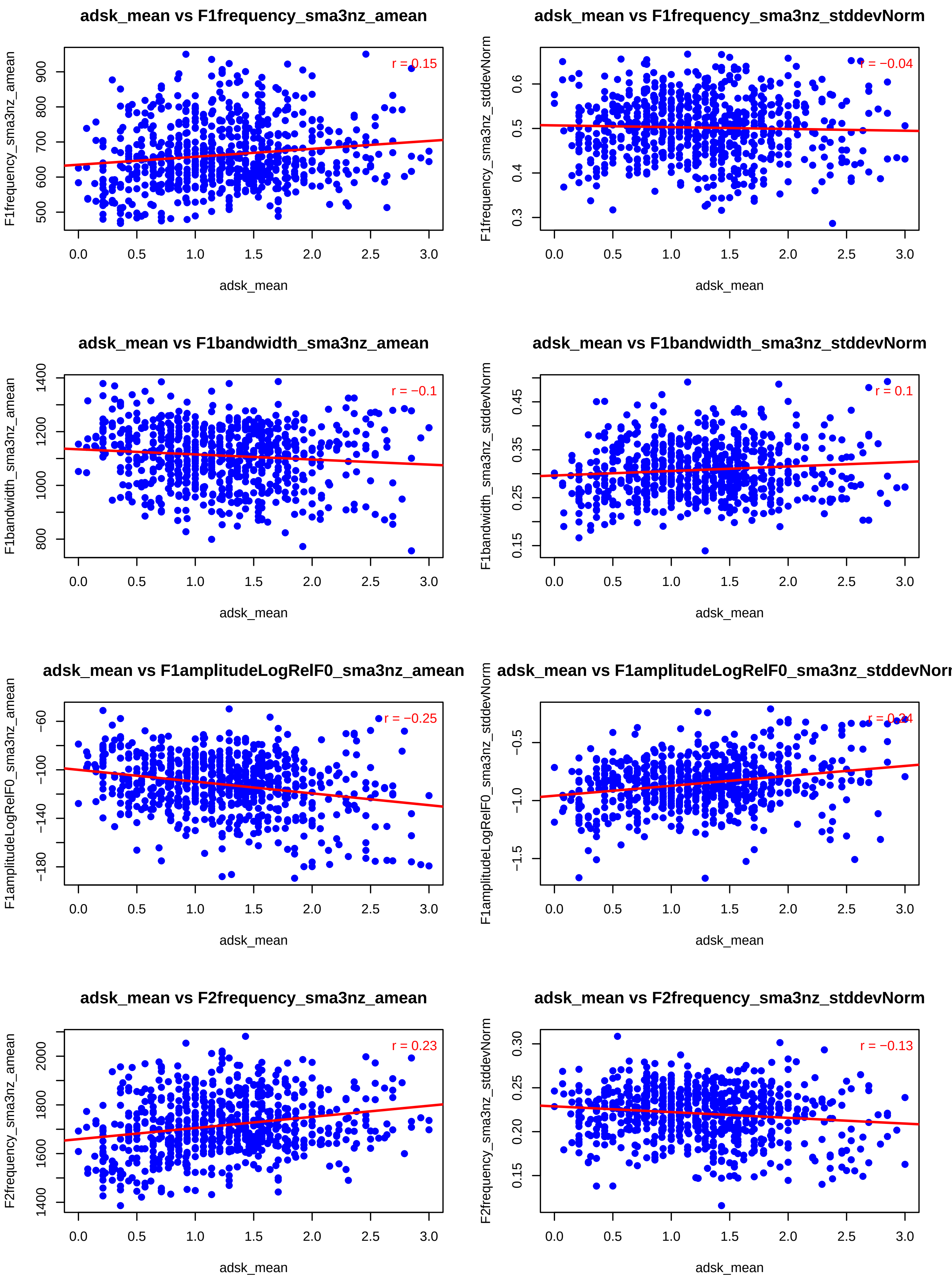
adsk_mean vs F1frequency_sma3nz_amean
r = 0.15
F1frequency_sma3nz_amean
adsk_mean
adsk_mean vs F1frequency_sma3nz_stddevNorm
r = −0.04
F1frequency_sma3nz_stddevNorm
adsk_mean
adsk_mean vs F1bandwidth_sma3nz_amean
r = −0.1
F1bandwidth_sma3nz_amean
adsk_mean
adsk_mean vs F1bandwidth_sma3nz_stddevNorm
r = 0.1
F1bandwidth_sma3nz_stddevNorm
adsk_mean
adsk_mean vs F1amplitudeLogRelF0_sma3nz_amean
r = −0.25
F1amplitudeLogRelF0_sma3nz_amean
adsk_mean
adsk_mean vs F1amplitudeLogRelF0_sma3nz_stddevNorm
r = 0.24
F1amplitudeLogRelF0_sma3nz_stddevNorm
adsk_mean
adsk_mean vs F2frequency_sma3nz_amean
r = 0.23
F2frequency_sma3nz_amean
adsk_mean
adsk_mean vs F2frequency_sma3nz_stddevNorm
r = −0.13
F2frequency_sma3nz_stddevNorm
adsk_mean

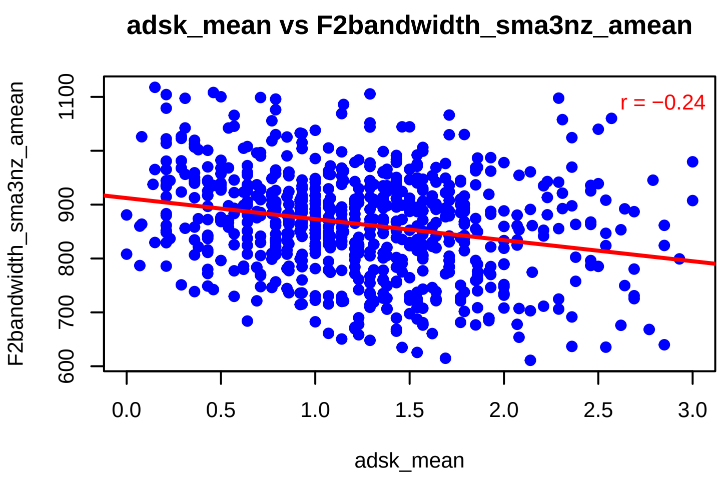
adsk_mean vs F2bandwidth_sma3nz_amean
r = −0.24
F2bandwidth_sma3nz_amean
adsk_mean

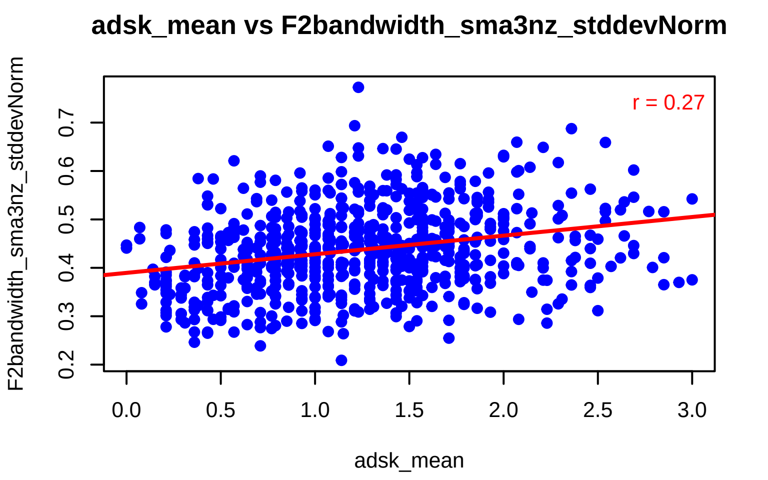
adsk_mean vs F2bandwidth_sma3nz_stddevNorm
r = 0.27
F2bandwidth_sma3nz_stddevNorm
adsk_mean

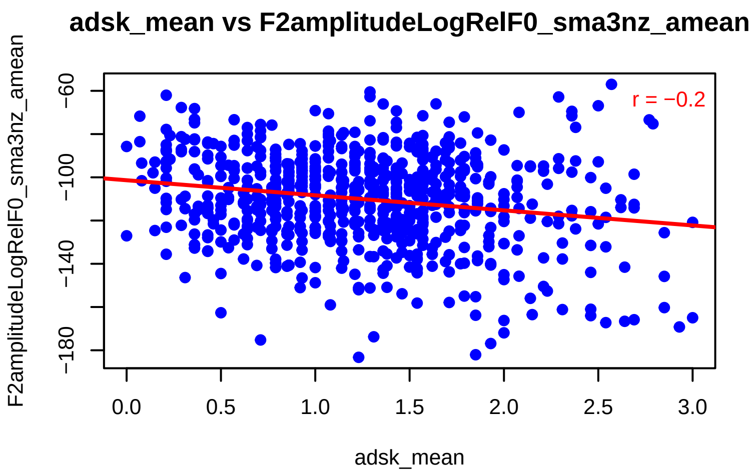
adsk_mean vs F2amplitudeLogRelF0_sma3nz_amean
r = −0.2
F2amplitudeLogRelF0_sma3nz_amean
adsk_mean

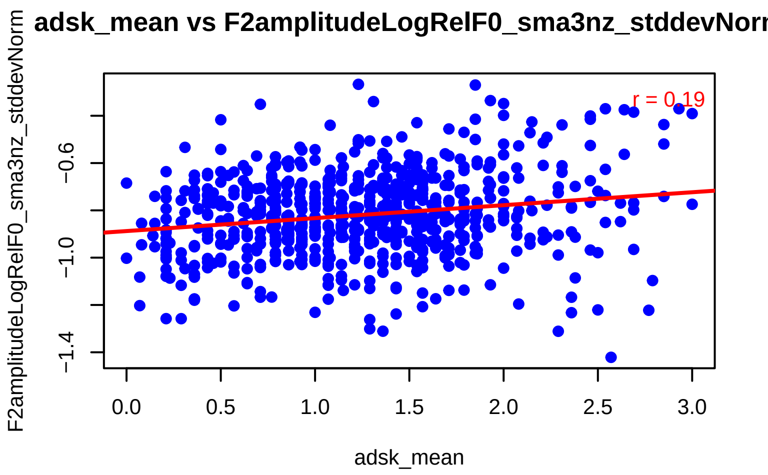
adsk_mean vs F2amplitudeLogRelF0_sma3nz_stddevNorr
r = 0.19
F2amplitudeLogRelF0_sma3nz_stddevNorm
adsk_mean

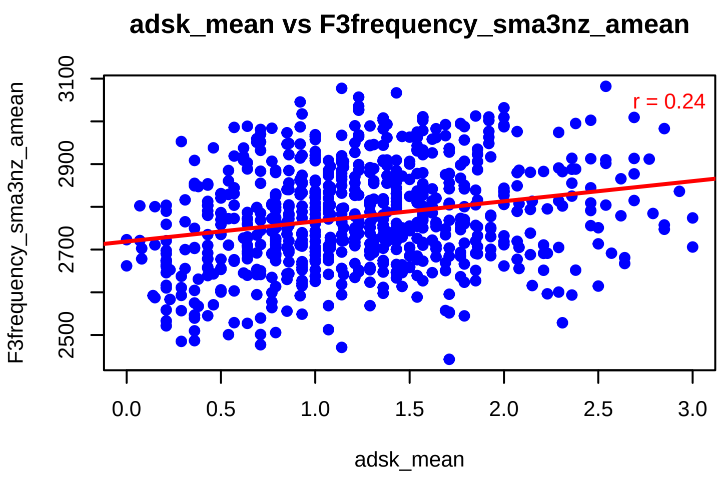
adsk_mean vs F3frequency_sma3nz_amean
r = 0.24
F3frequency_sma3nz_amean
adsk_mean

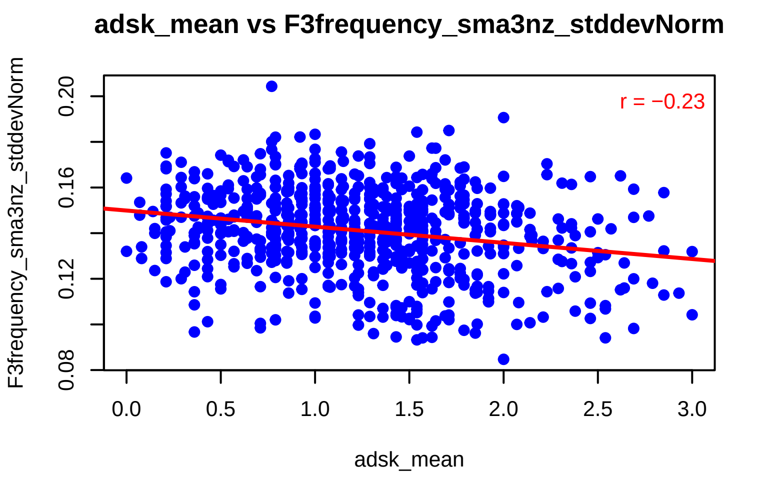
adsk_mean vs F3frequency_sma3nz_stddevNorm
r = −0.23
F3frequency_sma3nz_stddevNorm
adsk_mean

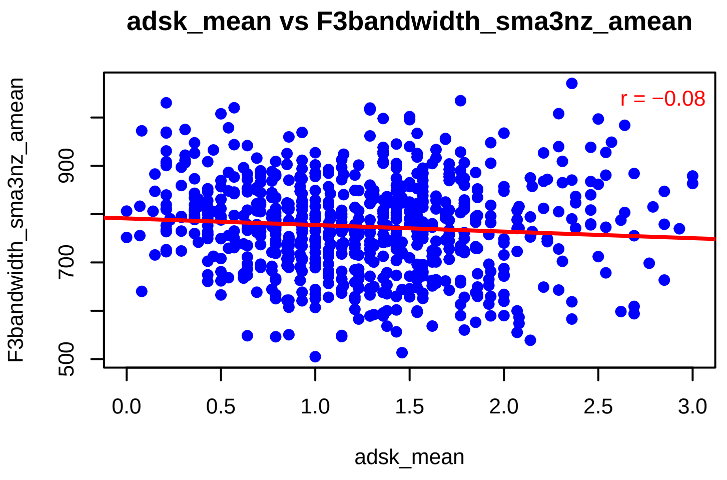
adsk_mean vs F3bandwidth_sma3nz_amean
r = −0.08
F3bandwidth_sma3nz_amean
adsk_mean

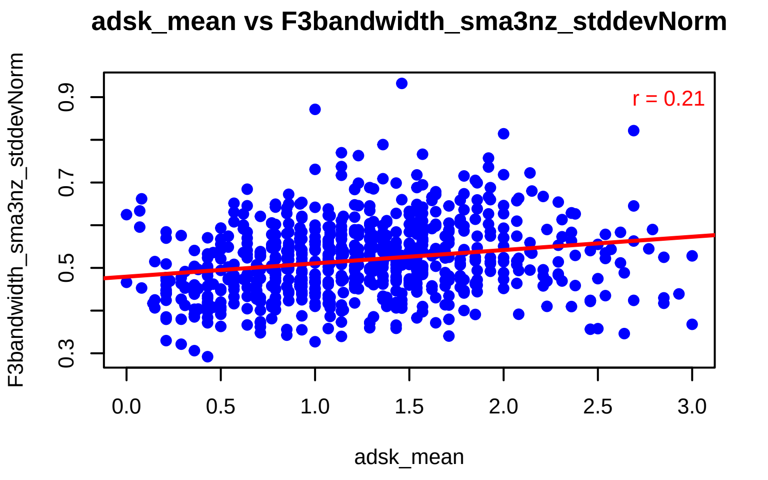
adsk_mean vs F3bandwidth_sma3nz_stddevNorm
r = 0.21
F3bandwidth_sma3nz_stddevNorm
adsk_mean

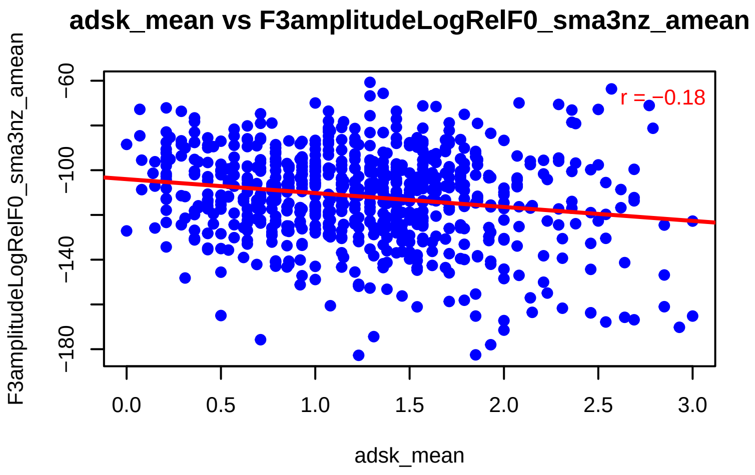
adsk_mean vs F3amplitudeLogRelF0_sma3nz_amean
r = −0.18
F3amplitudeLogRelF0_sma3nz_amean
adsk_mean

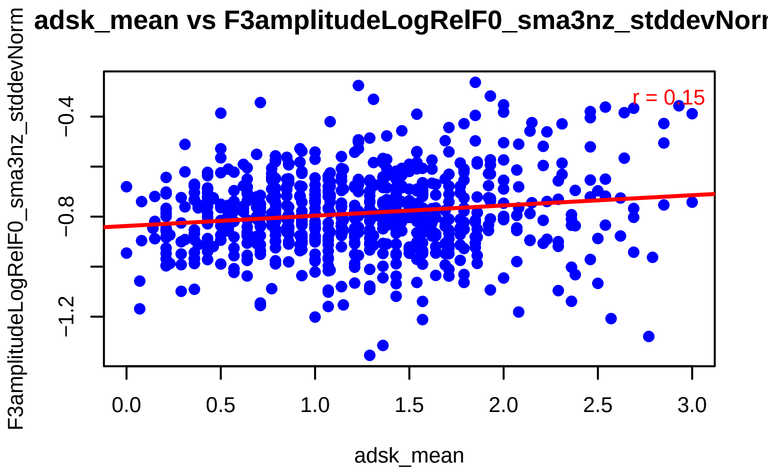
adsk_mean vs F3amplitudeLogRelF0_sma3nz_stddevNor
r = 0.15
F3amplitudeLogRelF0_sma3nz_stddevNorm
adsk_mean

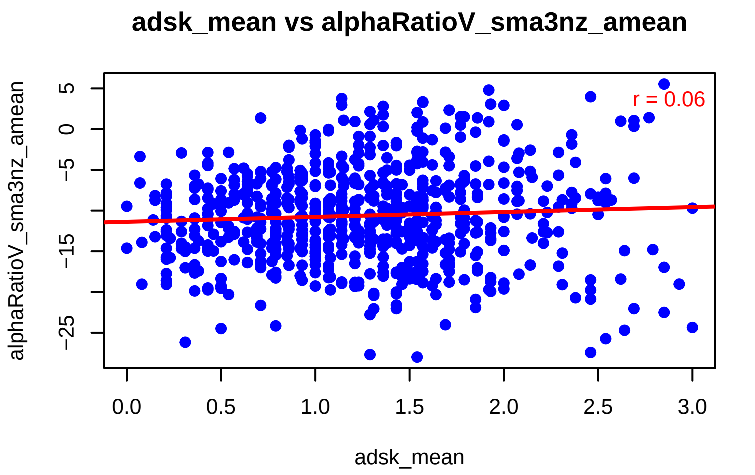
adsk_mean vs alphaRatioV_sma3nz_amean
r = 0.06
alphaRatioV_sma3nz_amean
adsk_mean

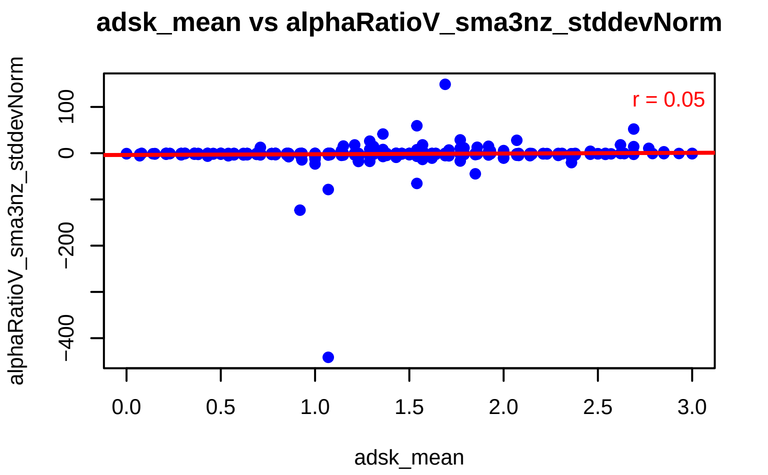
adsk_mean vs alphaRatioV_sma3nz_stddevNorm
r = 0.05
alphaRatioV_sma3nz_stddevNorm
adsk_mean

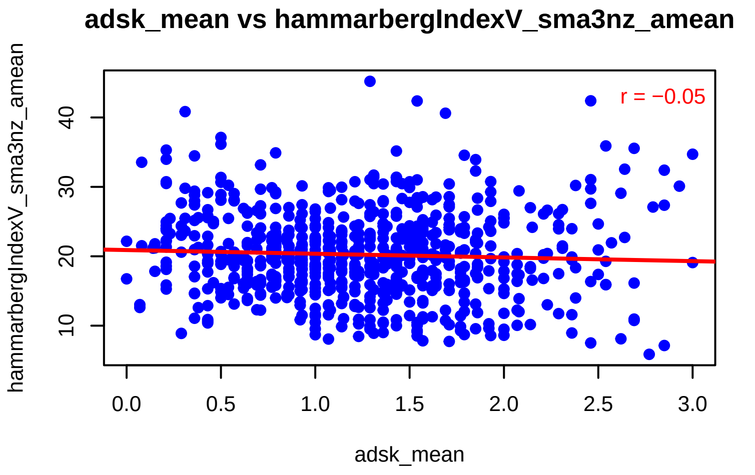
adsk_mean vs hammarbergIndexV_sma3nz_amean
r = −0.05
hammarbergIndexV_sma3nz_amean
adsk_mean

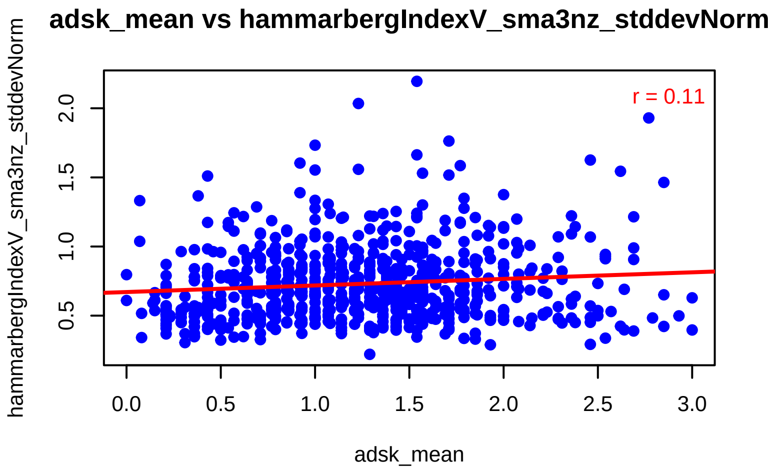
adsk_mean vs hammarbergIndexV_sma3nz_stddevNorm
r = 0.11
hammarbergIndexV_sma3nz_stddevNorm
adsk_mean

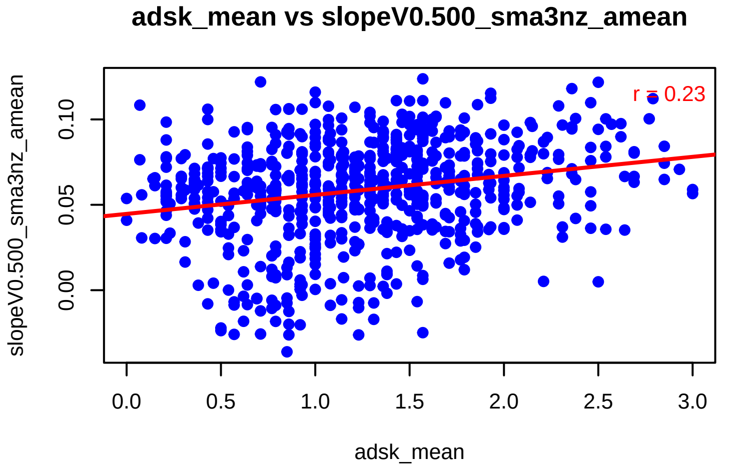
adsk_mean vs slopeV0.500_sma3nz_amean
r = 0.23
slopeV0.500_sma3nz_amean
adsk_mean

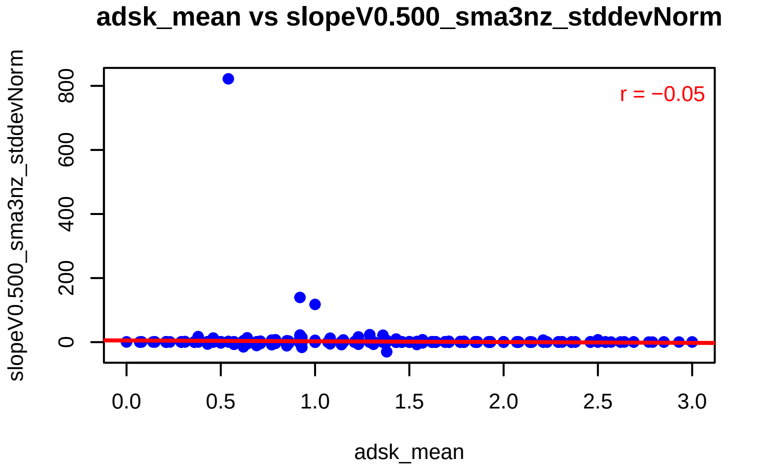
adsk_mean vs slopeV0.500_sma3nz_stddevNorm
r = −0.05
slopeV0.500_sma3nz_stddevNorm
adsk_mean

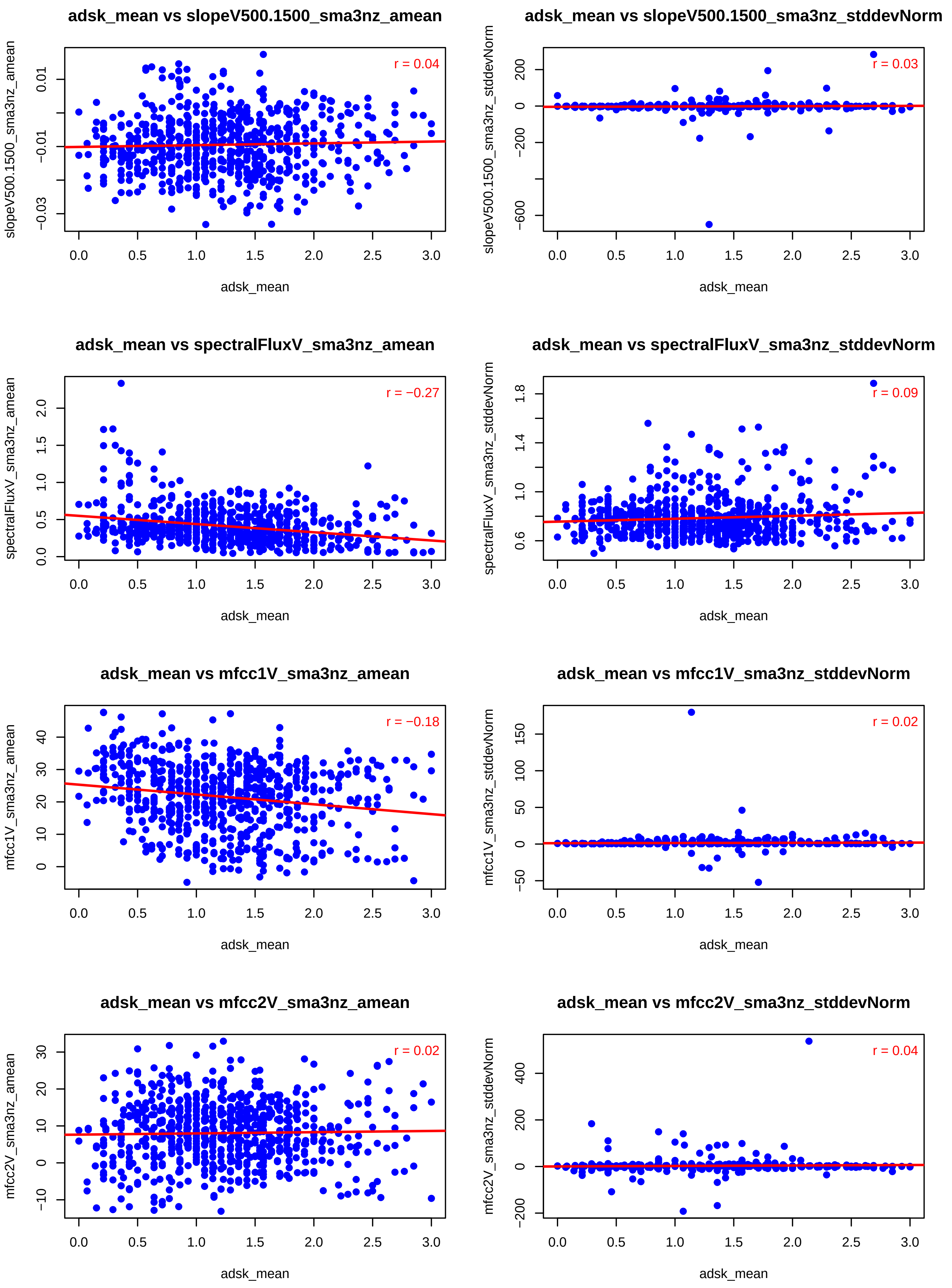
adsk_mean vs slopeV500.1500_sma3nz_amean
r = 0.04
slopeV500.1500_sma3nz_amean
adsk_mean
adsk_mean vs slopeV500.1500_sma3nz_stddevNorm
r = 0.03
slopeV500.1500_sma3nz_stddevNorm
adsk_mean
adsk_mean vs spectralFluxV_sma3nz_amean
r = −0.27
spectralFluxV_sma3nz_amean
adsk_mean
adsk_mean vs spectralFluxV_sma3nz_stddevNorm
r = 0.09
spectralFluxV_sma3nz_stddevNorm
adsk_mean
adsk_mean vs mfcc1V_sma3nz_amean
r = −0.18
mfcc1V_sma3nz_amean
adsk_mean
adsk_mean vs mfcc1V_sma3nz_stddevNorm
r = 0.02
mfcc1V_sma3nz_stddevNorm
adsk_mean
adsk_mean vs mfcc2V_sma3nz_amean
r = 0.02
mfcc2V_sma3nz_amean
adsk_mean
adsk_mean vs mfcc2V_sma3nz_stddevNorm
r = 0.04
mfcc2V_sma3nz_stddevNorm
adsk_mean

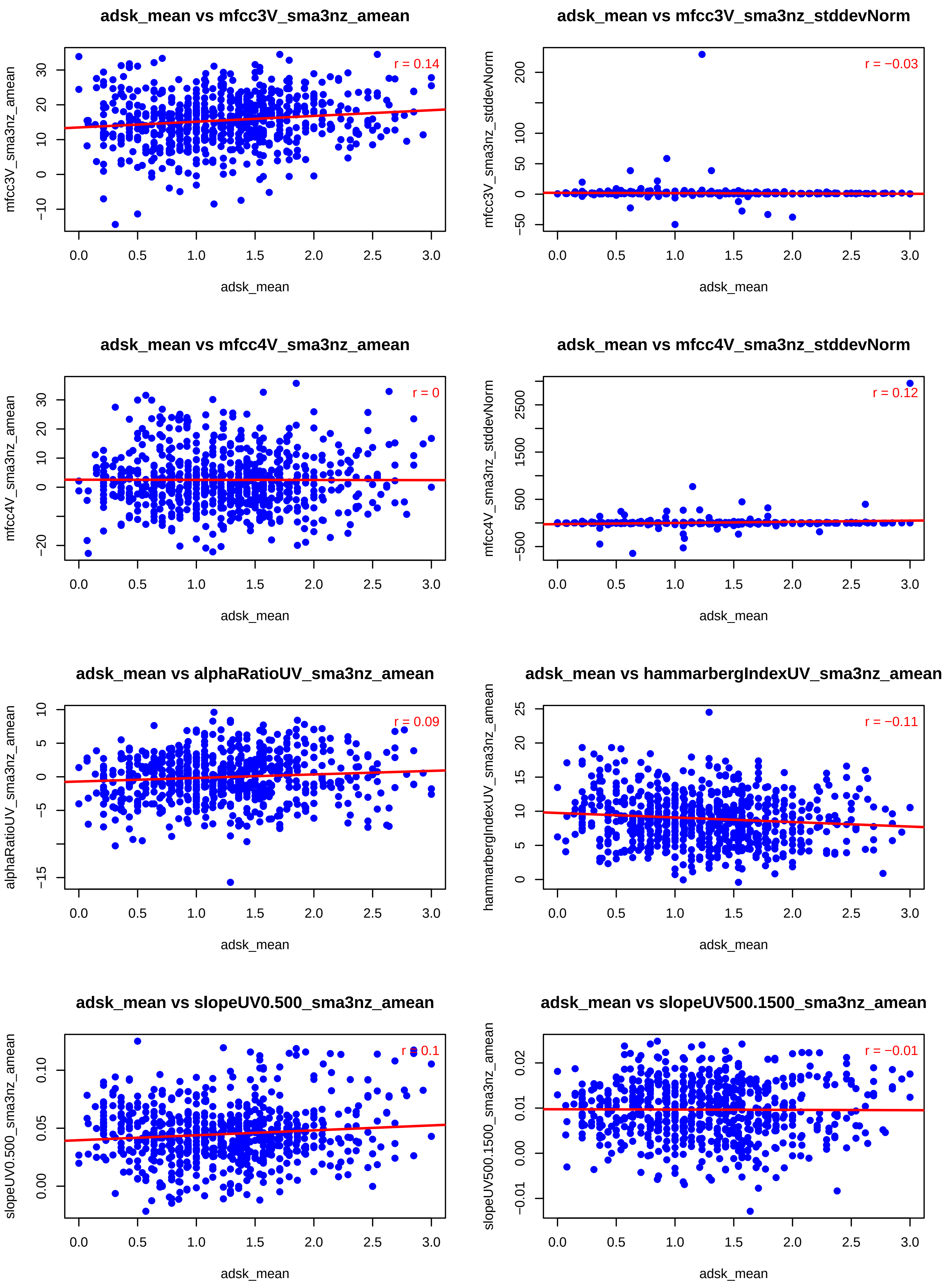
adsk_mean vs mfcc3V_sma3nz_amean
r = 0.14
mfcc3V_sma3nz_amean
adsk_mean
adsk_mean vs mfcc3V_sma3nz_stddevNorm
r = −0.03
mfcc3V_sma3nz_stddevNorm
adsk_mean
adsk_mean vs mfcc4V_sma3nz_amean
r = 0
mfcc4V_sma3nz_amean
adsk_mean
adsk_mean vs mfcc4V_sma3nz_stddevNorm
r = 0.12
mfcc4V_sma3nz_stddevNorm
adsk_mean
adsk_mean vs alphaRatioUV_sma3nz_amean
r = 0.09
alphaRatioUV_sma3nz_amean
adsk_mean
adsk_mean vs hammarbergIndexUV_sma3nz_amean
r = −0.11
hammarbergIndexUV_sma3nz_amean
adsk_mean
adsk_mean vs slopeUV0.500_sma3nz_amean
r = 0.1
slopeUV0.500_sma3nz_amean
adsk_mean
adsk_mean vs slopeUV500.1500_sma3nz_amean
r = −0.01
slopeUV500.1500_sma3nz_amean
adsk_mean

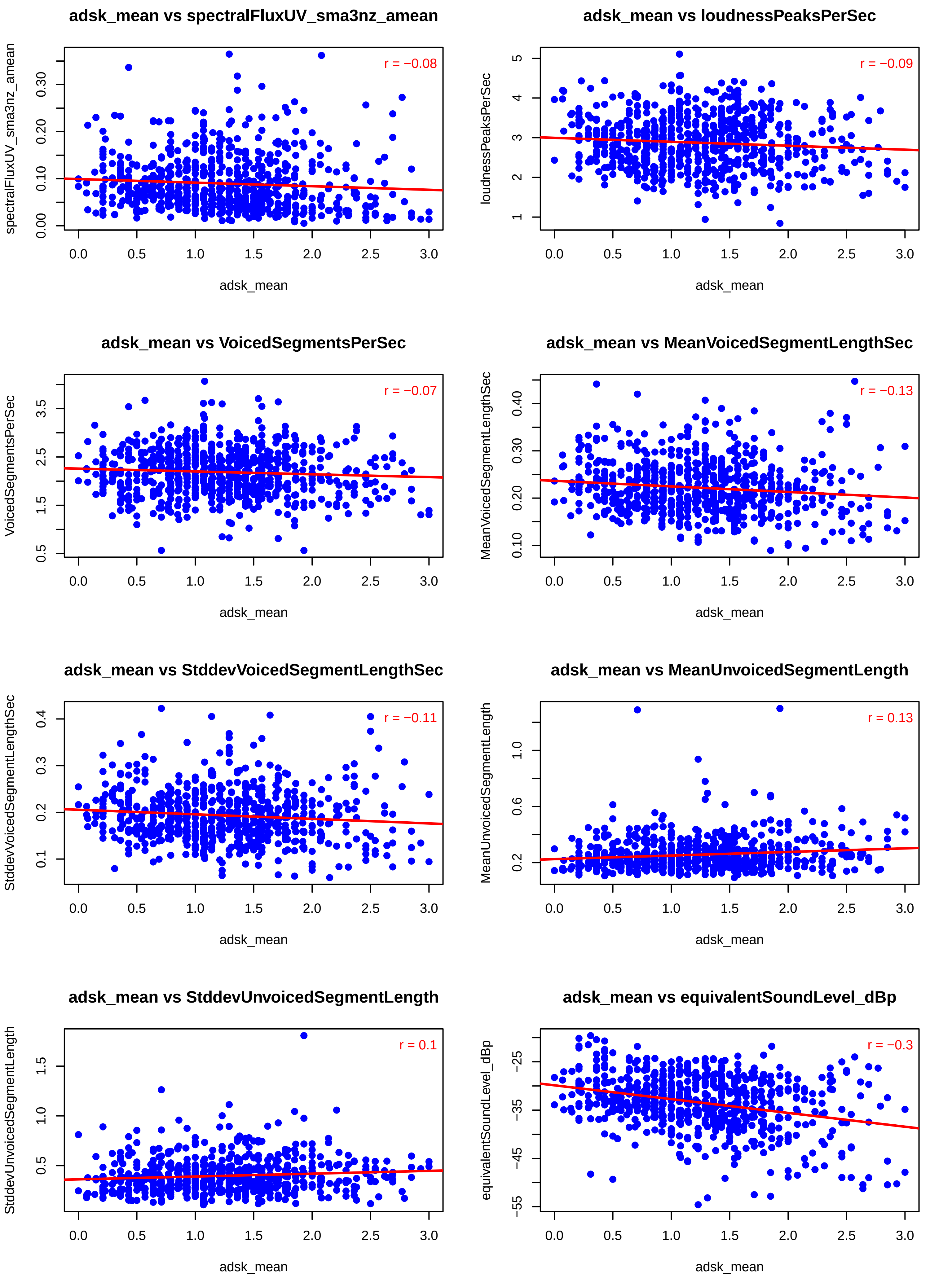
adsk_mean vs spectralFluxUV_sma3nz_amean
r = −0.08
spectralFluxUV_sma3nz_amean
adsk_mean
adsk_mean vs loudnessPeaksPerSec
r = −0.09
loudnessPeaksPerSec
adsk_mean
adsk_mean vs VoicedSegmentsPerSec
r = −0.07
VoicedSegmentsPerSec
adsk_mean
adsk_mean vs MeanVoicedSegmentLengthSec
r = −0.13
MeanVoicedSegmentLengthSec
adsk_mean
adsk_mean vs StddevVoicedSegmentLengthSec
r = −0.11
StddevVoicedSegmentLengthSec
adsk_mean
adsk_mean vs MeanUnvoicedSegmentLength
r = 0.13
MeanUnvoicedSegmentLength
adsk_mean
adsk_mean vs StddevUnvoicedSegmentLength
r = 0.1
StddevUnvoicedSegmentLength
adsk_mean
adsk_mean vs equivalentSoundLevel_dBp
r = −0.3
equivalentSoundLevel_dBp
adsk_mean

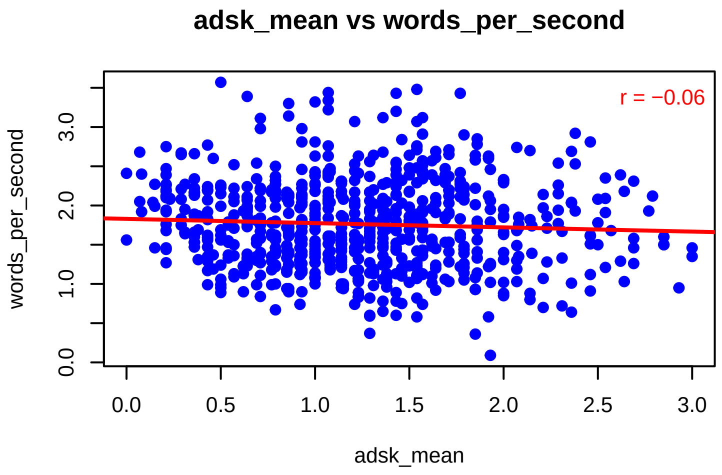

adsk_mean vs words_per_second
r = −0.06
words_per_second
0.0
1.0
2.0
3.0
0.0
0.5
1.0
1.5
2.0
2.5
3.0
adsk_mean
